# Hepatocyte-Specific Transcriptional Responses to Liver-Targeted Delivery of a Soluble Epoxide Hydrolase Inhibitor in a Mouse Model of Alcohol-Associated Liver Disease

**DOI:** 10.3390/biology14091267

**Published:** 2025-09-13

**Authors:** Dennis R. Warner, Jeffrey B. Warner, Josiah E. Hardesty, Yasmeen Abdelfadil, Chirag Soni, Philip Bauer, Claudio Maldonado, Craig J. McClain, Irina A. Kirpich

**Affiliations:** 1 Division of Gastroenterology, Hepatology, and Nutrition, Department of Medicine, University of Louisville, Louisville, KY 40202, USA; dennis.warner@louisville.edu (D.R.W.);; 2 Department of Pharmacology and Toxicology, University of Louisville, Louisville, KY 40202, USA; 3 Department of Microbiology and Immunology, University of Louisville, Louisville, KY 40202, USA; 4 Endo ProTech, Louisville, KY 40202, USA; 5 Department of Physiology, University of Louisville, Louisville, KY 40202, USA; 6 University of Louisville Alcohol Center, University of Louisville, Louisville, KY 40202, USA; 7 University of Louisville Hepatobiology & Toxicology Center, University of Louisville, Louisville, KY 40202, USA; 8 Robley Rex Veterans Medical Center, Louisville, KY 40202, USA

**Keywords:** alcohol-associated liver disease, soluble epoxide hydrolase, liposomes, liver specific delivery

## Abstract

Alcohol-associated liver disease (ALD) is a chronic condition caused by excessive alcohol consumption, with limited effective pharmacological treatments currently available. The inhibition of soluble epoxide hydrolase (s-EH) has emerged as a promising therapeutic strategy in experimental ALD. In this study, we developed a novel liver-targeted formulation of the s-EH inhibitor *t*-TUCB, encapsulated in fusogenic lipid vesicles (FLVs), and tested its therapeutic efficacy in a mouse model of ALD. This formulation achieved high encapsulation efficiency and targeted primarily hepatocytes. Male C57BL/6J mice were fed an ethanol-containing liquid diet for 10 days, followed by a single ethanol binge on day 11. A subset of mice received a single intraperitoneal dose of *t*-TUCB-FLVs two hours before the binge. *t*-TUCB-FLVs significantly attenuated liver injury as assessed by multiple endpoints, including decreased plasma ALT levels and hepatocyte cell death. Spatial transcriptomic analysis revealed that *t*-TUCB-FLV treatment modulated gene expression in hepatocytes across multiple key pathways, including xenobiotic metabolism, carbohydrate and lipid metabolism, bile acid homeostasis, inflammation, energy balance, and circadian rhythm. Collectively, these findings support liver-specific s-EH inhibition as a potentially effective therapeutic approach for ALD.

## Simple Summary

Alcohol-associated liver disease (ALD) remains a serious global health challenge, with limited effective treatments beyond alcohol abstinence. In this study, we evaluated a novel, liver-targeted therapy using *t*-TUCB, a compound with the potential to enhance the liver’s protective pathways. In a commonly used mouse model of ALD, delivering a single dose of *t*-TUCB to the liver significantly reduced alcohol-induced liver damage. These findings support *t*-TUCB as a promising candidate for future development of effective treatments for ALD.

## 1. Introduction

Excessive alcohol consumption is associated with a broad spectrum of adverse health effects across multiple organs, with the liver being particularly susceptible due to its central role in ethanol (EtOH) metabolism. The hepatic metabolism of EtOH produces toxic intermediate products such as acetaldehyde, a reactive compound that can form adducts with proteins and DNA, triggering oxidative stress, mitochondrial injury, and the activation of programmed cell death pathways, which are recognized as central mechanisms for the development of alcohol-associated liver disease (ALD) (reviewed in [1]). ALD encompasses a range of conditions, from simple steatosis (characterized by fat accumulation in the liver) to steatohepatitis (characterized by fat accumulation and inflammation), fibrosis, cirrhosis and, eventually, hepatocellular carcinoma [2]. Currently, the molecular mechanisms driving ALD progression are not fully understood, and effective therapies are lacking for all stages of the disease. This highlights an unfulfilled need for novel therapeutic targets and strategies to improve outcomes in ALD.

Recent studies from our group [3,4] and others [5] have demonstrated that the pharmacological inhibition or genetic ablation of the enzyme soluble epoxide hydrolase (s-EH) attenuated liver injury in animal models that recapitulate human ALD at both early stages, characterized by mild hepatic injury, and at more advanced stages, such as alcohol-associated hepatitis. s-EH is a cytosolic enzyme that reduces the levels of cytochrome P-450 (CYP)-generated epoxy fatty acids (Ep-FAs) derived from n6 and n3 polyunsaturated fatty acids (PUFAs) by converting these lipid mediators into inactive or less active diols (dihydroxy-FAs). Multiple Ep-FAs, known for their anti-inflammatory properties [6,7], can promote the resolution of inflammation [8], and tissue/organ regeneration (e.g., the liver) [9]. s-EH has recently emerged as a promising therapeutic target for several other diseases beyond ALD. Indeed, pharmacological s-EH inhibition or genetic s-EH ablation in animal models has shown therapeutic potential in various pathological conditions, including inflammation, pain, cancer, and kidney disease, to name a few [10,11,12,13,14,15,16,17]. Additionally, the beneficial effects of s-EH genetic deficiency or pharmacological inhibition have also been shown in various animal models of liver disease, including diet-induced hepatic steatosis, metabolic dysfunction-associated steatohepatitis (MASH), carbon-tetrachloride (CCl_4_)-induced liver fibrosis, and a partial hepatectomy model [18,19,20,21] (reviewed in [22]).

Key mechanisms underlying the protective effects of systemic s-EH inhibition in ALD involve both hepatic and extrahepatic processes. In the liver, s-EH inhibition reduced EtOH-induced oxidative stress and endoplasmic reticulum stress, which are key contributors to hepatocellular injury, as well as attenuation of both local and systemic inflammation [3,4,5]. Beyond the liver, systemic s-EH inhibition was associated with changes in the gut microbiota composition, characterized by an increase in beneficial bacterial taxa and attenuation of EtOH-induced microbial imbalances, which likely contributed to improved gut barrier function further mitigating hepatic inflammation and injury [3]. To gain a more comprehensive understanding of the liver-specific effects of s-EH inhibition, independent of its potential systemic actions, the objectives of the current study were to develop a liver-targeted delivery system for an s-EH inhibitor, evaluate its therapeutic efficacy in a well-characterized mouse model of ALD, and investigate the underlying mechanisms of action, with a focus on hepatocyte transcriptional responses.

## 2. Materials and Methods

### 2.1. Preparation of t-TUCB Fusogenic Lipid Vesicles (t-TUCB-FLVs)

To develop a liver-targeted delivery platform for s-EH inhibition, we encapsulated the s-EH inhibitor, 4-[[trans-4-[[[[4-Trifluoromethoxy) phenyl] amino] carbonyl]-amino] cyclohexyl] oxy]-benzoic acid (*t*-TUCB) into fusogenic lipid vesicles, thus generating *t*-TUCB-FLVs. Although, there are several s-EH inhibitors available for use in animal studies, we selected *t*-TUCB due to its high effectiveness in inhibiting s-EH in mice (IC_50_ = 5.7 nM [23]), and based upon previous studies demonstrating its beneficial effects in preclinical models of liver pathologies, including portal hypertension [24], fibrosis [25], and ALD, as have been reported recently in our own studies [3,4]. To prepare *t*-TUCB-FLVs, a 10 mg/mL of a lipid solution consisting of 1,2-dioleoyl-sn-glycero-3-phosphocholine (DOPC) and 1-palmitoyl-2-oleol-sn-glycerol-3-phosphate (POPA, both from Avanti Polar Lipids, Alabaster, AL, USA) at a 3:2 molar ratio was prepared; this was then mixed with a 10.3 mg/mL solution of *t*-TUCB in EtOH to achieve a final concentration of 3.0 mg/mL of *t*-TUCB. Heat–vortex cycles were used to thoroughly encapsulate *t*-TUCB into the lipid formulation. The solution was sonicated, then centrifuged at 1000× *g* for 10 min. The supernatant was extruded through membranes with decreasing pore sizes of 400 nm, 200 nm, and 100 nm, consecutively. FLVs were analyzed (diameter and zeta potential) using a ZetaView nanoparticle tracking analyzer instrument (Particle Metrix, Ammersee, Germany). Before administration to mice, the *t*-TUCB-FLV solution was filter-sterilized and a portion was labeled with 10 µg/mL of the far-red fluorescent, lipophilic carbocyanine, DiD (Thermo Fisher, Waltham, MA, USA) to determine *t*-TUCB-FLV tissue/cell localization by fluorescence imaging.

### 2.2. Animal Studies

Male C57BL/6J mice were purchased from the Jackson Laboratory (Bar Harbor, ME, USA) and housed in a specific pathogen-free animal facility accredited by the Association for Assessment and Accreditation of Laboratory Animal Care. The room was maintained on a 12 h light/dark cycle. The study was approved by the University of Louisville Institutional Animal Care and Use Committee. A well-established model of ALD that we have previously employed [4], which recapitulates advanced ALD in humans such as early stage of alcohol-associated hepatitis, was utilized in the current study. Specifically, 8- to 10-week-old mice (6–10/group) were provided a 5% (*v*/*v*) EtOH-containing all-liquid Lieber–DeCarli diet for 10 days followed by a single EtOH “binge” on day 11, delivered by oral gavage (5 g/kg) 9 h before euthanasia. Two hours prior to the EtOH binge, a subset of mice were administered either 3 mg/kg *t*-TUCB-FLVs by intraperitoneal injection (i.p.), or “empty” FLVs as a control. Following treatment with *t*-TUCB and the EtOH gavage, mice continued to have free access to the liquid diet. The dose of 3 mg/kg *t*-TUCB was selected based upon our pilot studies wherein we evaluated a range of *t*-TUCB-FLV doses (0.3–9 mg/kg) to establish the optimal concentration for this animal model. We found that 3 mg/kg *t*-TUCB-FLVs provided the greatest protection from EtOH-induced liver injury as assessed by plasma ALT levels. The time of *t*-TUCB-FLV treatment, specifically 2 h prior to the EtOH binge, was chosen based on the kinetics of FLV uptake by the liver as determined in a previous study using FLVs of a similar formulation (30). That study demonstrated that labeled FLVs were localized primarily to the liver with peak detection at 2 h post-injection.

At the termination of the experiment, mice were deeply anesthetized with ketamine/xylazine and blood was collected from the inferior vena cava into heparinized microcentrifuge tubes. The plasma fraction was prepared by centrifugation at 2000× *g* for 10 min, aliquoted, and stored at −80 °C for future analyses. Portions of liver tissue from the left hepatic lobe were snap-frozen in liquid nitrogen and stored at −80 °C, placed in Optimum Cutting Temperature (OCT) media (Sakura Finetek, Torrance, CA, USA) and stored at −80 °C, or fixed in 10% neutral buffered formalin (Fisher Scientific, Waltham, MA, USA) for embedding in paraffin for histological assessments.

### 2.3. Assessment of Liver Damage

Alanine aminotransferase (ALT) activity, as a marker of liver injury, was determined using the ALT/GPT reagent (Thermo Fisher). Formalin-fixed, paraffin-embedded liver sections were stained with hematoxylin and eosin (H&E) for gross morphological assessment. Terminal deoxynucleotidyl transferase dUTP nick end labeling (TUNEL) staining was performed for the analysis of hepatocyte cell death using the ApopTag Peroxidase In Situ Apoptosis Detection Kit (Sigma Chemical Co., St. Louis, MO, USA). Chloroacetate esterase (CAE, Sigma Chemical) staining was performed for assessment of liver neutrophil infiltration. TUNEL- and CAE-positive cells were counted in 10 random (200× total magnification digital microscope) fields per liver section by an investigator blinded to the experimental groups.

### 2.4. Measurement of Plasma EtOH Concentration

The concentration of EtOH in plasma samples was measured using the EnzyChrom Ethanol Assay Kit according to the manufacturer’s instructions (BioAssay Systems, Hayward, CA, USA).

### 2.5. t-TUCB-FLV Tissue and Cell Distribution Visualization

To assess tissue localization of *t*-TUCB-FLVs, representative DID-labeled OCT-embedded liver and kidney sections were visualized using the Cy5 channel in a Keyence BZ-X800 All-In-One Fluorescence Microscope (Keyence, Elmwood Park, NJ, USA). In order to determine *t*-TUCB-FLV cellular distribution, representative fresh liver sections were homogenized using the gentle MACS Tissue Dissociator (Miltenyi Biotec, Gaithersburg, MD, USA). The resulting homogenates were subjected to magnetic-assisted cell sorting to isolate specific cell populations, including hepatocytes, macrophages, and lymphocytes. DiD fluorescence, indicating FLV uptake, was measured in each cell population via flow cytometry using a BD FACS Canto II instrument coupled to BD FACS Diva software (BD, Franklin Lakes, NJ, USA) with beads alone as a negative control.

### 2.6. Liver Lipidomics and Triglyceride (TG) Analyses

Liver-targeted lipidomic analysis was performed on snap-frozen liver tissue by the Wayne State University Lipidomic Core Facility using standard practices established in the Facility. For assessment of TGs, lipids were extracted from liver tissue with chloroform and methanol as previously described [26] and TGs were measured using Infinity™ reagents from Thermo Fisher according to the manufacturers’ instructions.

### 2.7. NanoString Liver Spatial Transcriptomics Analysis

Formalin-fixed, paraffin-embedded liver sections were cut to a thickness of 5 µm and mounted to the center of Superfrost Plus precleaned microscope slides (Fisher Scientific), then provided to the NanoString Digital Spatial Profiling (DSP) Technology Access Program (Seattle, WA, USA). To prepare the slides for DSP, liver tissue sections (n = 1 per each group) were stained against Pan-Cytokeratin to segment for hepatocytes. Three areas of interest (AOI) were manually selected as technical replicates under a fluorescence microscope. Immunofluorescence, region/AOI selection/segmentation, and barcode cleavage and collection were performed using the NanoString GeoMX DSP platform.

For each AOI, Whole Transcriptome Atlas (WTA) datasets were passed through QC with default settings, then filtered and Q3 normalized using the GeoMX DSP Analysis Suite version 2.5.1.145 to produce the final datasets for analysis. A one-way ANOVA was performed across all treatment groups and a post hoc Tukey test was performed for groupwise comparisons. The Benjamini–Hochberg procedure was applied to generate adjusted *p*-values to control for the false discovery rate. R Studio version 4.4.2 was used for statistical analysis. Bioinformatics was performed with Gene Set Enrichment Analysis (GSEA [27,28]) and g:Profiler [29]. The GSEA approach included all genes in the dataset regardless of whether they are significantly changed or not in order to detect subtle shifts that may not be detected by focusing on individual genes that meet specific criteria (e.g., fold-change and *p*-value cutoff).

### 2.8. Statistical Analysis

Data are shown as the mean ± standard error of the mean (SEM). GraphPad Prism 9.3.1 software (Boston, MA, USA) was used to analyze data other than NanoString transcriptomics by performing one-way ANOVA with Sidak’s multi-comparison test for data following a normal distribution and Mann–Whitney and Kruskal–Wallis test with Dunn’s multi-comparison test for data following a non-normal distribution. Data were considered significantly different at *p* < 0.05. Heatmaps and PCA plots for lipidomic data were created in MetaboAnalyst freeware (https://www.metaboanalyst.ca; accessed on 6 June 2025).

## 3. Results

### 3.1. Characterization of a Liver-Targeted s-EH Inhibitor Delivery Platform

**Figure 1:**
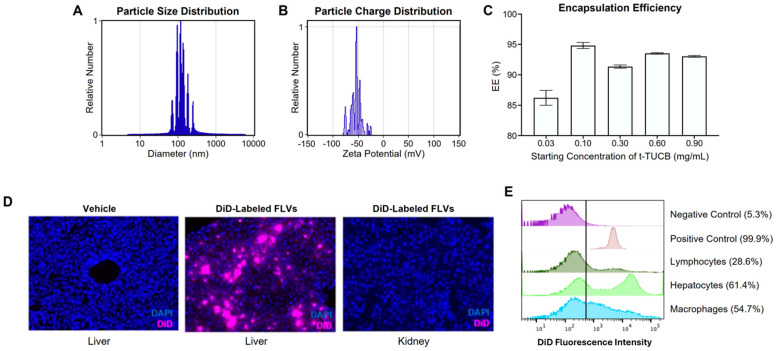
Development and characterization of the *t*-TUCB-FLV platform. (**A**) FLV particle size distribution (in nm). (**B**) FLV charge distribution (zeta potential, mV). (**C**) Encapsulation efficiency of *t*-TUCB into FLVs. (**D**) Fluorescence microscopy of tissue cryosections from mice injected with unlabeled FLVs (liver) or DID-labeled FLVs (liver and kidney) and counterstained with DAPI. (**E**) Analysis of the cellular distribution of FLVs in the liver of mice injected with DID-labeled FLVs, expressed as fluorescence intensity of DiD. Negative control was hepatocytes isolated from mouse injected with unlabeled FLVs and the positive control was DiD-labeled FLVs. The vertical line represents the threshold fluorescence indicating a positive signal. The percentages indicate fraction of signal above the threshold.

In the current study, we explored a strategy for precision targeting of the liver parenchyma with *t*-TUCB, utilizing a *t*-TUCB-FLV platform which passively targets *t*-TUCB to the liver and cell cytoplasm while escaping endosomal degradation. Previous studies employing this FLV platform, which is composed of DOPC and POPA, demonstrated the tropism of the FLVs for the liver in live imaging studies [30]. In our study, the mean diameter and zeta potential of *t*-TUCB-FLV preparation were 130 nm and −55 mV (Figure 1A and Figure 1B, respectively), which are the optimal physical properties of FLVs for passive liver targeting. Preparations of *t*-TUCB-FLVs consistently achieved an encapsulation efficiency of 85–90% (Figure 1C). To confirm tissue localization, the lipophilic fluorescent marker DiD was incorporated into *t*-TUCB-loaded nanoparticles. Following i.p. administration of 3 mg/kg DiD-labeled *t*-TUCB-FLVs, liposomes were detected in the liver (Figure 1D), whereas non-treated controls and non-targeted tissues (e.g., kidney) showed no fluorescence signal. Further, DiD fluorescence using flow cytometric analysis on various liver cell populations revealed that hepatocytes, and to a lesser extent macrophages, were the primary cell types taking up the labeled *t*-TUCB-FLVs (Figure 1E).

### 3.2. Effects of t-TUCB-FLVs on an Animal Model of ALD

**Figure 2:**
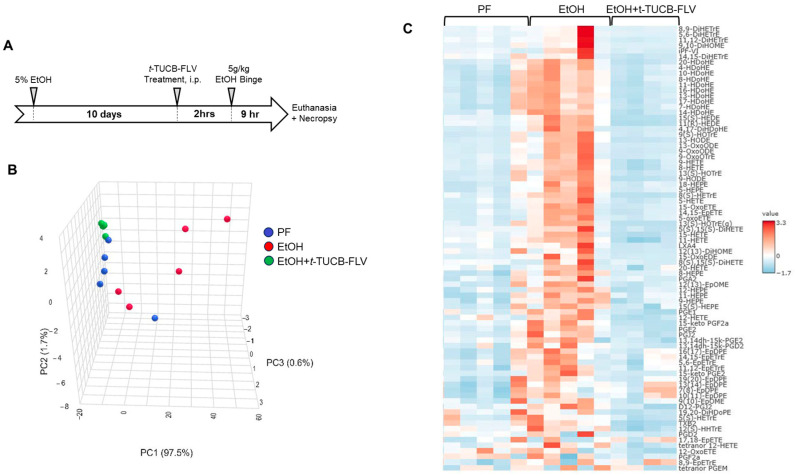
Hepatic lipidomic changes mediated by EtOH and *t*-TUCB-FLVs in a mouse model of ALD. (**A**) Experimental animal model. (**B**) PCA plot of lipidomic changes in PF, EtOH-fed, and *t*-TUCB-FLV-treated EtOH-fed mice. (**C**) Heat map of the relative expression of hepatic lipid molecules.

To test the *t*-TUCB liver-specific delivery platform, *t*-TUCB-FLVs, and evaluate its therapeutic efficacy, we utilized a well-established murine model of ALD. Mice were fed an EtOH-containing diet for 10 days, followed by a single EtOH binge (by oral gavage) on day 11, administered 9 h prior to euthanasia. *t*-TUCB-FLVs were delivered in a treatment paradigm as a single i.p. dose of 3 mg/kg *t*-TUCB-FLVs or vehicle control (“empty” FLVs) administered 2 h before the EtOH binge (Figure 2A).

#### 3.2.1. *t*-TUCB-FLVs Induce Significant Changes in the Hepatic Lipidomic Profile

Given that s-EH inhibition is known to modulate the pools of Ep-FAs, we performed liver targeted lipidomic analysis. Principal component analysis (PCA) revealed that EtOH-fed mice treated with *t*-TUCB-FLVs exhibited a distinct lipidomic signature compared to untreated EtOH-fed controls (Figure 2B). A heat map depicting the levels of all quantified lipid molecules further illustrates distinct lipid profiles among PF, EtOH-fed and EtOH + *t*-TUCB-FLV-treated mice (Figure 2C). Comparisons of individual lipid metabolites among all experimental groups are presented Table 1. As an indirect measure of s-EH activity, the ratios of multiple epoxide-to-diol pairs (Ep-FA:DhFA) were calculated. Compared to EtOH-fed mice, those treated with EtOH + *t*-TUCB-FLV exhibited a significant increase in several individual ratios, including n3 PUFA-derived metabolites such as the DHA-derived 19(20)-EpDPE:DiHDoPE, as well as n6 PUFA-derived metabolites including the arachidonic acid-derived 5(6)-EpETrE:DiHETrE, 8(9)-EpETrE:DiHETrE, and 14(15)-EpETrE:DiHETrE, along with the linoleic acid-derived, 9(10)-EpOME:DiHOME pair. The cumulative elevation in these individual ratios resulted in a significant increase in the overall Ep-FA:DhFA ratio in EtOH + *t*-TUCB-FLV group (Table 1).

#### 3.2.2. *t*-TUCB-FLVs Attenuated EtOH-Induced Liver Damage

**Figure 3:**
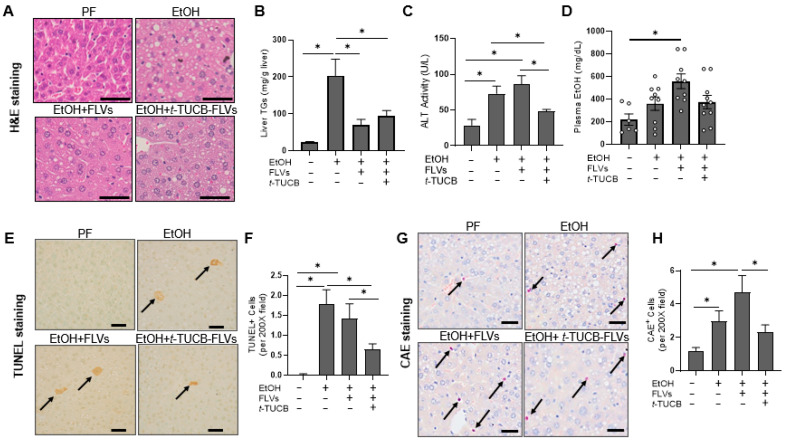
Effects of t-TUCB-FLVs on EtOH-induced liver damage in an animal model of ALD. (**A**) Representative image of H&E-stained liver sections demonstrating gross morphology. Scale bar is 50 µm. (**B**) Liver triglyceride levels. (**C**) Plasma ALT levels. (**D**) Plasma EtOH levels. (**E**) Representative TUNEL-stained liver sections. Arrows indicate TUNEL-positive cells. Scale bar is 50 µm. (**F**) Quantitation of TUNEL-positive cells from 10 random fields per section. (**G**) Representative CAE-stained sections. Arrows indicate CAE-positive cells. Scale bar is 50 µm. (**H**) Quantitation of CAE-positive cells from 10 random fields per section. Data are presented as the mean ± SEM and statistical significance determined by one-way ANOVA with Sidak’s multi-comparison test; * *p* < 0.05.

EtOH-induced hepatic steatosis was significantly decreased by both *t*-TUCB-FLVs and empty FLV controls, as evident from the H&E-stained liver sections (Figure 3A) and hepatic triglyceride measurement (Figure 3B). These findings suggest that FLVs alone may modulate liver fat accumulation, with a limited effect by *t*-TUCB. However, *t*-TUCB-FLV treatment significantly decreased EtOH-induced plasma ALT levels, an indirect marker of liver injury, while this protective effect was not observed in EtOH-fed mice receiving empty FLVs (Figure 3C). Consumption of the EtOH-containing diet resulted in an increase in plasma EtOH levels in EtOH-fed and EtOH+FLV-treated mice (Figure 3D). There were no significant differences between EtOH-fed and EtOH+*t*-TUCB-FLV-treated mice, suggesting that the mechanism by which *t*-TUCB-FLVs attenuated EtOH-induced liver damage was not attributed to the differences in EtOH levels or metabolism. To assess the extent of hepatocyte cell death, a hallmark of EtOH toxicity [31] and a common feature of ALD, liver tissue sections were evaluated using the TUNEL assay. EtOH significantly increased the number of TUNEL-positive cells in the liver, while *t*-TUCB-FLVs, but not FLV controls notably reduced their numbers in EtOH-fed mice (Figure 3E,F). Additionally, liver CAE staining (a marker of neutrophil accumulation) showed a significant increase in neutrophil numbers in the EtOH-fed group (Figure 3G,H), which was alleviated by *t*-TUCB-FLVs. Of note, similar to ALT results, empty FLV controls had no effects on EtOH-induced increases in TUNEL^+^ or CAE^+^ cells, indicating that empty FLVs have minimal impact on EtOH-induced liver injury. Taken together, while both *t*-TUCB-FLVs and empty FLVs ameliorated EtOH-induced hepatic steatosis, the hepatoprotective effects appeared specific to *t*-TUCB-FLVs, suggesting the therapeutic potential of *t*-TUCB in mitigating EtOH-induced liver injury but not steatosis. The limited effects of *t*-TUCB on EtOH-induced liver steatosis have been previously observed by us in experimental models of ALD using systemic *t*-TUCB administration [3,4].

### 3.3. Hepatocyte-Specific Transcriptional Responses to EtOH and t-TUCB-FLVs

**Figure 4:**
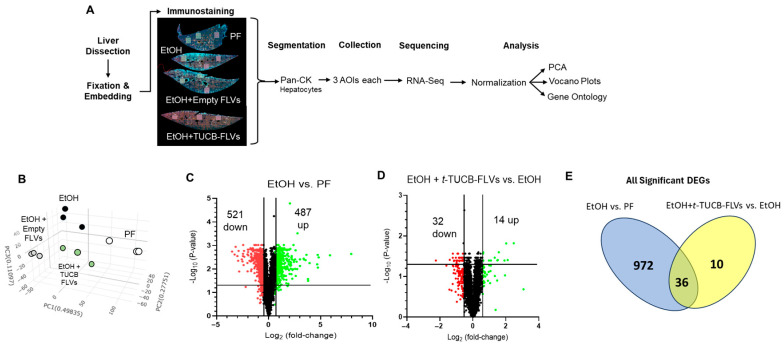
Spatial liver transcriptomic analysis. (**A**) Outline of the experimental approach to measure differential gene expression mediated by *t*-TUCB-FLV treatment in hepatocytes. (**B**) PCA plot. (**C**) Volcano plot for genes with altered expression by EtOH. (**D**) Volcano plot for gene expression changes in response to *t*-TUCB-FLVs in EtOH-fed mice. Plots illustrate up (green) or down (red)-regulated genes with a minimum fold-change of 1.5 and *p*-value of <0.05 (indicated by the vertical and horizontal lines, respectively. (**E**) Venn diagram of significant DEGs regulated by EtOH (blue) and *t*-TUCB-FLVs in EtOH-fed mice (yellow).

To investigate potential molecular mechanisms underlying the beneficial effects of *t*-TUCB on EtOH-induced liver injury, we performed spatial transcriptomic analysis using the GeoMx Digital Spatial Profiler platform (NanoString Technologies, Seattle, WA, USA). This approach, summarized in Figure 4A, enabled high-resolution, location-specific gene expression profiling within liver tissue, allowing us to identify cell-specific, particularly hepatocyte-specific, responses to *t*-TUCB treatment. We focused on the effects of *t*-TUCB on hepatocytes because these cells were the primary targets of *t*-TUCB-FLVs (Figure 1E), and play a central role in EtOH detoxification, and intracellular signaling to other, nonparenchymal cells, including hepatic stellate cells [32] and immune cells [33]. PCA revealed distinct clustering patterns of hepatocyte transcriptomes across all experimental groups (Figure 4B). As anticipated, EtOH feeding resulted in a clear separation from PF control mice, reflecting substantial transcriptional reprogramming induced by EtOH exposure. Of note, hepatocytes from mice treated with *t*-TUCB-FLVs and FLV control also formed separate clusters, both from each other and from the EtOH group. This distinction suggests that *t*-TUCB-FLVs and FLVs each elicit unique transcriptional responses, and that *t*-TUCB-FLVs may confer effects beyond the FLV control. Thus, as compared to PF controls, EtOH-fed mice had 487 up-regulated genes compared to 521 down-regulated genes in hepatocytes that met the criteria of at least a 1.5-fold change and adjusted *p*-value < 0.05 (Figure 4C). The complete gene list is provided in Supplemental Table S1. *t*-TUCB-FLV treatment of EtOH-fed mice led to 14- and 32-up and down-regulated genes, respectively (Figure 4D).

To better understand the impact of *t*-TUCB-FLVs on hepatocyte gene expression in the context of EtOH exposure, we analyzed the overlap between genes regulated by EtOH and those altered by *t*-TUCB-FLVs in EtOH-fed mice. A total of 1008 genes were differentially expressed in the EtOH vs. PF comparison, while 46 genes were significantly altered in the EtOH + t-TUCB-FLV vs. EtOH comparison. Among these, 10 genes were uniquely regulated by *t*-TUCB-FLVs and were not significantly affected by EtOH alone, and 36 genes overlapped between the two comparisons, as shown in the Venn diagram (Figure 4E). Further analysis revealed that *t*-TUCB-FLVs reversed the expression of 28 of these 36 overlapping genes. Specifically, 21 genes that were upregulated by EtOH were downregulated by *t*-TUCB-FLVs, and 7 genes that were suppressed by EtOH were upregulated following *t*-TUCB-FLV treatment (Table 2). The remaining 8 overlapping genes showed consistent directionality of change across both comparisons; however, their magnitude of change was more pronounced in the EtOH vs. PF group.

**Figure 5:**
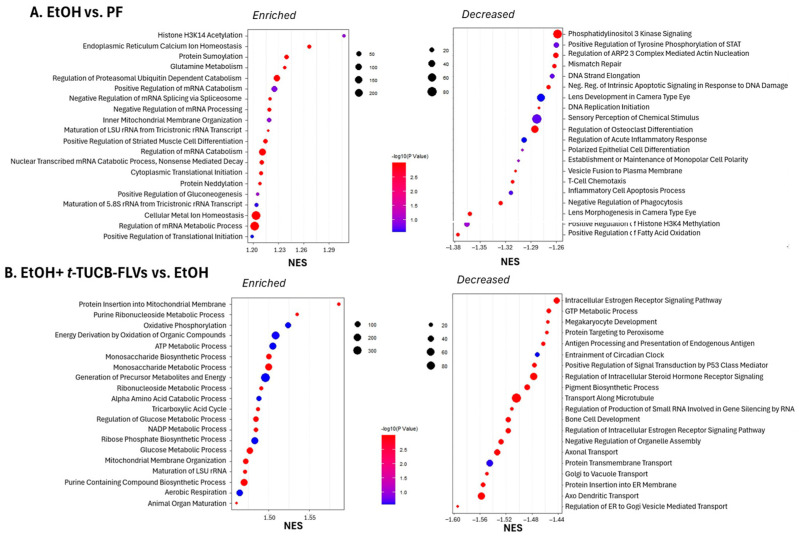
Bubble plots of gene set enrichment analysis (GSEA) of the hepatocyte transcriptome. (**A**) Comparison of PF vs. EtOH-fed mice. (**B**) Comparison of EtOH+*t*-TUCB-FLV treated vs. EtOH-fed mice. Pathways shown are either significantly enriched or decreased. The size of the bubbles indicates the number of genes (Counts), and the color intensity indicates statistical significance (−log10 *p* value). NES, normalized enrichment score.

Functional analysis performed by Gene Set Enrichment Analysis [27,28], which includes all expressed genes, regardless of statistical significance, revealed that EtOH exposure led to enrichment of pathways related to histone acetylation, protein modification (including sumoylation, ubiquitination, and neddylation), and mRNA processing, among others. In contrast, pathways associated with PI3K signaling, apoptosis, regulation of inflammation, and fatty acid oxidation were downregulated in EtOH-fed mice (Figure 5A). Treatment with *t*-TUCB-FLVs resulted in enriched pathways involved in peroxisome function, ER membrane protein targeting, protein transport, and cell signaling (including p53 and steroid hormone signaling). Conversely, *t*-TUCB-FLVs suppressed pathways related to oxidative phosphorylation, amino acid metabolism, ribonucleoside metabolism, glucose metabolism, and NADP metabolism, among others (Figure 5B). Collectively, these findings suggest that both EtOH and *t*-TUCB-FLVs modulate a broad array of molecular pathways, which may underline the harmful hepatic effects of EtOH and the protective actions of *t*-TUCB-FLVs. We next focused on the bioinformatic analysis of genes significantly regulated by *t*-TUCB-FLVs in EtOH-treated mice. We performed gene ontology (GO) enrichment analysis using g:Profiler [29] which clustered these into biologically meaningful categories, including bile acid synthesis and metabolism (*Errfi1*, *Cyp7b1*, and *Ces1d*), regulation of circadian rhythm (*Egr1*, *Klf10*, *Npas2*, *Cyp7b1*, *Ces1D*, *Hes6*), and ion transport (*Arl6ip5*, *Slc10a2*, *Slc39a14*, *Slc7a2*, *SlcO1a1*, and *Ces1d*) (Table 3).

## 4. Discussion

In this study, we developed a liver-specific delivery system for the s-EH inhibitor, *t*-TUCB, using fusogenic lipid vesicles (FLVs) as a novel approach to target the liver. The physicochemical properties of *t*-TUCB supported its formulation into liposomes: it is poorly soluble in water [34] but compatible with liposomal systems, which can encapsulate both hydrophilic and lipophilic compounds. Our liposomal preparations achieved encapsulation efficiencies exceeding 85–90%, underscoring the practicality and potential of this delivery platform. This *t*-TUCB-FLV platform efficiently delivered *t*-TUCB to the liver, with hepatocytes as the primary target cells. Targeted drug delivery has long been explored by researchers as a strategy to enhance therapeutic efficacy, minimize systemic toxicity, and optimize pharmacokinetics in the treatment of liver diseases, including ALD, as we have previously reviewed [35]. A variety of nanomedicine platforms, such as liposomes, polymeric nanoparticles, exosomes, engineered bacteria, and adeno-associated virus vectors, have been employed to deliver a broad spectrum of therapeutic agents in preclinical ALD models. These agents include phosphodiesterase inhibitors [30], microRNAs [36], enzymes [37], and anti-inflammatory cytokines [38], among others. By enabling cell-specific or organ-specific delivery, these platforms have shown improved targeting of hepatic cells, enhanced bioavailability, and greater therapeutic benefit in rodent models.

A key finding of our study was that *t*-TUCB-FLVs conferred therapeutic benefits in a mouse model of ALD. The beneficial effect of s-EH inhibition observed here is consistent with previous studies using genetic *Ephx2* ablation or various s-EH inhibitors in murine models of ALD [3,4,5]. However, unlike earlier studies that primarily employed systemic s-EH inhibition in a preventive setting, our study demonstrated that a single dose of *t*-TUCB-FLVs targeted to the liver in a treatment paradigm significantly attenuated ALD. An added advantage of this approach is the reduction in systemic exposure and the minimization of potential off-target effects in other organs. Although we, and to the best of our knowledge, others, did not observe overt adverse effects of systemic pharmacological s-EH inhibition on animal health and behavior, there still exists the possibility of off-target effects. In animals, systemic ablation of s-EH has been associated with unintended effects in whole-body *Ephx2* knockout models, such as reduced plasma testosterone, sperm count, and testicular size in males [39], as well as decreased serum estradiol in females treated with an s-EH inhibitor [40]. Some evidence suggests minor adverse events, such as headache and contact dermatitis as possible side effects of the s-EH inhibitor, GSK2256294, in humans [41]. Therefore, targeted, organ-specific s-EH inhibitor delivery may help mitigate the risk of systemic side effects. Additionally, delivery of s-EH inhibitors in particular to the liver may also allow for lower effective doses while enhancing therapeutic efficacy of s-EH inhibitors in patients with liver disease. In the current study, *t*-TUCB-FLVs were administered to mice by i.p. injection; however, this approach has limited clinical translatability for long-term therapy. One promising alternative could be the use of orally administered edible exosomes (*e.g.* ginger-derived nanoparticles), which have been previously shown to selectively target the liver, predominantly hepatocytes, [42]. Such systems could provide a non-invasive route of administration, and enable sustained, organ-specific delivery of s-EH inhibitors. Further studies in this direction would be a next significant step toward translating the efficacy observed in the current study into a clinically relevant strategy for chronic liver disease management.

Based on our observation that *t*-TUCB-FLVs primarily interacted with hepatocytes, we employed a cell type-targeted transcriptomic approach using the GeoMX platform to specifically examine the effects of *t*-TUCB-FLVs on EtOH-mediated gene expression changes in hepatocytes. Many of the genes differentially expressed in EtOH and *t*-TUCB-FLV-treated mice showed expression changes that opposed the effects of EtOH, suggesting a reversal of EtOH-induced transcriptional alterations by *t*-TUCB-FLVs. An example among these proteins was *Npas2*, whose expression was significantly increased by EtOH, but reduced by *t*-TUCB-FLV treatment. NPAS2, a paralog of the CLOCK protein, interacts with BMAL1 to regulate the expression of key proteins involved in the circadian rhythm [43]. Disruption of circadian rhythm has been recognized among the major factors contributing to the harmful effects of alcohol on the liver (reviewed in [44]). Beyond its role in circadian regulation, NPAS2 has also been implicated in promoting liver fibrosis through its regulation of *Hes1* expression [45]. Another gene involved in the circadian rhythm, *Hes6*, was also differentially regulated by EtOH (down-regulated) and *t*-TUCB-FLVs (up-regulated). *Hes6* plays a complementary role in circadian regulation by antagonizing the expression of *Hes1* [46] and has been shown to attenuate hepatic steatosis [47]. These findings suggest that *t*-TUCB-FLV-mediated restoration of *Hes6*, along with suppression of *Npas2* and *Hes1*, may help reverse circadian dysregulation and simultaneously mitigate fibrotic and metabolic dysfunction in ALD. Another prominent gene was *Egr1*, whose expression was decreased to a greater extent by *t*-TUCBs-FLVs than other genes and was one of the most highly up-regulated by EtOH alone. EGR1 is a transcription factor that regulates the expression of numerous genes, including many important for liver health, including genes regulating fibrosis [48], inflammation [49] and EtOH-induced hepatic steatosis [50].

Of the genes differentially regulated by *t*-TUCB-FLVs in EtOH-fed mice, 10 were unique to *t*-TUCB-FLVs (i.e., not also differentially regulated by EtOH) and all but two of these (*Erdr1* and *Gmppa*) were down-regulated. Functional analysis of several of these genes reveals possible roles in ameliorating liver diseases, including ALD. For example, it has been previously shown that the expression of *Erdr1* is up-regulated in M2 macrophages [51], which would be predicted to attenuate EtOH-induced chronic inflammation. It has been shown that downregulation of *Txnip* expression attenuates activation of hepatic stellate cells [52]. *Txnip* also has other functions including binding and inhibiting the antioxidant protein, thioredoxin, activation of the NLRP3 inflammasome, and mediates ER-stress activation of apoptosis (reviewed in [53]). Indeed, *Txnip* has become an attractive target for drug development due to its involvement in these pathways. Previous work has demonstrated that the gene, *Arrdc3*,modulates insulin action and glucose metabolism in the liver [54], which may be important for metabolic disorders, including metabolic dysfunction-associated steatotic liver disease. In addition, Yang et al. found that the expression of *Arrdc3* was significantly up-regulated in the livers of ALD patients and in mice fed EtOH, and that *Arrdc3* knockdown in mice ameliorated hepatic injury as revealed by reduced serum ALT and AST [55]. Lastly, *Anxa5* expression is upregulated early in the progression to hepatocellular carcinoma [56] and here we demonstrate that *t*-TUCB-FLVs decreased its expression, suggesting potential anti-cancer activities of s-EH inhibition. Therefore, *t*-TUCB-FLVs were able to modulate the expression of genes involved in several processes that may underly the mechanisms by which s-EH inhibition attenuates the disease pathology mediated by excessive alcohol consumption.

The current study should be considered in the context of its limitations. One of the limitations was that only male mice were used in this study. The inclusion of female mice is an important consideration for future studies as it has been previously demonstrated that female mice are more susceptible to ethanol-induced liver damage using the same NIAAA model that was used in our study [57]. In our single-cell spatial transcriptomics approach, we used only a biological n number of 1 per each experimental group. This type of analysis is relatively nascent, and thus, as costs drop, a higher biological n number may be more feasible. In light of this, we used AOIs across several zones of liver tissue in order to decrease spatial bias. Additionally, the specificity of the laser capture must be considered. While our spatial segmentation had a high degree of spatial resolution, contaminating transcripts may be present. Lastly, the WTA approach was largely descriptive, and identified trends associated with our treatment, including several potentially novel pathways; however, future studies will be required to validate these results and draw more mechanistic conclusions.

## 5. Conclusions

In summary, this study introduced a novel liver-targeted delivery system for the s-EH inhibitor, *t*-TUCB, achieving high encapsulation efficiency and specific hepatocyte targeting. In a mouse model of ALD, the liver-specific administration of *t*-TUCB-FLVs significantly alleviated liver injury. The observed reversal of EtOH-induced gene expression changes in hepatocytes suggests that *t*-TUCB-FLVs can restore disrupted cellular processes central to liver injury. Future studies will aim to further define the molecular pathways influenced by s-EH inhibition and expand this approach to explore the roles of non-parenchymal liver cells, such as Kupffer cells, hepatic stellate cells, and liver sinusoidal endothelial cells, in mediating the therapeutic effects of targeted s-EH inhibitor delivery. This will provide a more comprehensive understanding of the cellular crosstalk involved in ALD and may help identify additional therapeutic targets.

## Figures and Tables

**Table 1:** Hepatic oxylipin analysis.

	Lipid	EtOH vs. PF		EtOH+*t*-TUCB-FLV vs. EtOH	
		**Fold-Change**	***p*-Value**	**Fold-Change**	***p*-Value**
	PGE1	1.75	0.095	−4.19	**0.007**
	PGE2	2.53	**0.004**	−4.13	**0.001**
	15-keto PGE2	8.85	**0.012**	−25.32	**0.011**
	13,14dh-15k-PGE2	2.39	**<0.001**	−4.06	**<0.001**
	PGA2	1.67	0.603	−3.39	0.195
	tetranor PGEM	1.25	0.686	−1.33	0.596
	PGD2	1.81	0.178	−3.05	**0.049**
	PGJ2	2.32	0.066	−4.10	**0.022**
	D12-PGJ2	2.29	**0.025**	−3.20	**0.037**
	13,14dh-15k-PGD2	1.81	0.107	−2.29	0.053
	PGF2a	1.21	0.684	−1.93	0.107
	15-keto PGF2a	2.11	**0.015**	−4.22	**0.002**
	iPF-VI	5.11	0.216	−11.50	0.412
	TXB2	1.35	0.394	−5.63	**0.005**
	5(S),15(S)-DiHETE	2.80	0.145	−5.60	0.312
	8(S),15(S)-DiHETE	4.33	0.079	−10.11	0.084
	9-HODE	1.89	**0.043**	−3.54	**0.005**
	13-HODE	2.39	**0.033**	−4.70	**0.008**
	9(S)-HOTrE	3.64	**0.048**	−5.73	**0.034**
	13(S)-HOTrE	2.34	0.081	−4.89	**0.023**
	11(R)-HEDE	2.15	**0.005**	−5.67	**<0.001**
	15(S)-HEDE	2.54	**0.007**	−8.87	**0.001**
	8(S)-HETrE	2.54	0.010	−7.07	**0.001**
	5(S)-HETrE	1.19	0.865	−3.24	0.138
	5-HETE	2.92	0.034	−4.73	**0.018**
	8-HETE	1.83	0.023	−2.86	**0.004**
	9-HETE	2.12	0.008	−3.28	**0.002**
	11-HETE	1.38	0.007	−2.08	**<0.001**
	12-HETE	1.86	0.085	−4.92	**0.007**
	15-HETE	1.87	0.023	−3.69	**0.002**
	20-HETE	1.75	0.107	−2.01	0.072
	tetranor 12-HETE	2.04	0.178	−3.60	0.090
	12(S)-HHTrE	1.36	0.115	−2.30	**0.003**
	5-HEPE	4.64	0.008	−4.37	**0.013**
	8-HEPE	2.50	0.028	−2.56	**0.026**
	9-HEPE	2.14	**0.022**	−3.06	**0.006**
	11-HEPE	2.14	**0.010**	−1.83	**0.035**
	12-HEPE	3.16	**<0.001**	−3.50	**<0.001**
	15(S)-HEPE	2.00	0.116	−2.50	0.101
	18-HEPE	3.54	**0.006**	−3.25	**0.011**
	4-HDoHE	3.53	**0.004**	−4.04	**0.004**
	7-HDoHE	2.37	0.034	−3.24	**0.018**
	8-HDoHE	2.49	**0.012**	−2.95	**0.009**
	10-HDoHE	2.47	**0.009**	−3.33	**0.004**
	11-HDoHE	2.31	**0.015**	−3.45	**0.005**
	13-HDoHE	2.00	**0.031**	−2.89	**0.010**
	14-HDoHE	2.37	**0.009**	−4.09	**0.002**
	16-HDoHE	2.38	**0.008**	−3.39	**0.003**
	17-HDoHE	2.14	**0.033**	−3.53	**0.009**
	20-HDoHE	2.51	**0.005**	−4.46	**0.001**
**s-EH Substrates and Products**	9(10)-EpOME	1.38	0.731	−2.26	0.349
	12(13)-EpOME	2.13	0.175	−3.84	0.065
	5(6)-EpETrE	1.63	0.286	−1.84	0.222
	8(9)-EpETrE	1.79	0.320	−1.04	0.992
	11(12)-EpETrE	2.24	0.073	−2.23	0.075
	14(15)-EpETrE	1.91	0.159	−1.60	0.335
	14(15)-EpETE	5.00	**0.029**	−4.69	0.102
	17(18)-EpETE	1.17	0.958	−1.05	0.995
	7(8)-EpDPE	1.60	0.506	−0.88	0.923
	10(11)-EpDPE	1.86	0.329	−1.13	0.930
	13(14)-EpDPE	1.57	0.564	−1.03	0.997
	16(17)-EpDPE	2.58	0.113	−2.03	0.238
	19(20)-EpDPE	1.55	0.583	−2.00	0.401
	9(10)-DiHOME	3.78	0.345	−13.34	0.237
	12(13)-DiHOME	1.23	0.928	−7.76	0.280
	5(6)-DiHETrE	7.29	0.240	−18.83	0.220
	8(9)-DiHETrE	4.15	0.310	−9.97	0.240
	11(12)-DiHETrE	2.51	0.303	−5.97	0.150
	14(15)-DiHETrE	1.40	0.667	−3.82	0.129
	19(20)-DiHDoPE	−1.22	0.860	−4.18	0.243
	9-OxoODE	4.39	**0.046**	−14.54	**0.024**
	13-OxoODE	6.03	**0.038**	−16.41	**0.028**
	9-OxoOTrE	3.72	0.102	−8.87	0.059
	15-OxoEDE	5.95	0.072	−18.75	0.077
	5-oxoETE	7.58	**0.009**	−6.97	**0.014**
	12-OxoETE	1.03	0.994	−3.69	0.221
	15-OxoETE	4.41	**0.011**	−5.41	**0.011**
	LXA4	3.37	0.207	−13.40	0.373
	4,17-DiHDoHE	8.20	**0.017**	−17.57	**0.026**
	9(10)EpOME:DiHOME	−1.45	0.649	3.24	**0.003**
	12(13)EpOME:DiHOME	2.65	**0.028**	1.33	0.329
	5(6)-EpETrE:DiHETrE	−2.13	0.418	4.50	**0.006**
	8(9)-EpETrE:DiHETrE	−1.17	0.924	3.93	**<0.001**
	11(12)-EpETrE:DiHETrE	1.48	0.551	1.71	0.093
	14(15)-EpETrE:DiHETrE	1.90	0.149	1.73	**0.031**
	19(20)-EpDPE:DiHDoPE	2.70	**0.053**	1.73	**0.034**
	All Ep-FAs:Diols	1.03	0.995	1.83	**0.003**

Data are presented as fold-change and *p*-values calculated by one-way ANOVA. Numbers in bold are *p* < 0.05.

**Table 2:** Effect of *t*-TUCB-FLVs on hepatocyte gene expression.

	EtOH vs. PF		EtOH+*t*-TUCB FLV vs. EtOH	
Gene	log2FC	*p* Adj	log2FC	*p* Adj
Up-regulated by EtOH and Down-regulated by t-TUCB-FLVs				
Aass	2.086	0.001	−0.661	0.034
Arl6ip5	0.981	0.004	−0.675	0.040
Crcp	1.074	0.002	−0.678	0.040
Dop1b	1.170	0.006	−0.990	0.046
Egr1	3.553	0.004	−2.248	0.040
Errfi1	0.610	0.009	−0.689	0.034
Gm3776	1.039	0.005	−1.218	0.035
Gstt3	1.443	0.004	−1.013	0.040
Il1r1	1.897	0.001	−0.800	0.034
Klf10	1.169	0.004	−1.033	0.034
Mt2	7.955	0.002	−1.142	0.040
Npas2	0.701	0.011	−0.620	0.039
Pctp	1.247	0.001	−0.632	0.041
Ppp1r3b	0.900	0.002	−0.935	0.027
Sgk2	1.093	0.003	−0.791	0.040
Slc10a2	0.683	0.003	−0.787	0.034
Slc39a14	1.641	0.001	−0.893	0.027
Slc7a2	0.789	0.017	−0.916	0.040
Tm4sf4	1.100	0.003	−0.594	0.040
Tns1	0.722	0.002	−0.606	0.027
Zbtb16	0.919	0.004	−0.977	0.049
Down-regulated by EtOH and Up-regulated by t-TUCB-FLVs				
C6	−1.685	0.002	1.095	0.040
Cxcl12	−1.483	0.001	0.630	0.040
Cyp7b1	−1.111	0.008	1.918	0.040
Hes6	−1.465	0.004	0.630	0.040
Mup13	−2.027	0.002	1.517	0.039
Nudt7	−2.581	0.004	1.481	0.040
Slco1a1	−2.460	0.002	1.328	0.034
Regulated in same direction by EtOH and t-TUCB-FLVs				
Akr1c19	−1.024	0.010	−0.815	0.042
Apcs	2.781	<0.001	0.630	0.025
Ces1d	−0.746	0.004	−0.984	0.041
Fads2	−1.651	0.005	−0.655	0.034
Orm1	1.658	0.010	0.978	0.040
Orm2	1.759	0.002	2.112	0.039
Saa1	4.679	0.006	2.025	0.015
Saa2	4.743	0.009	2.481	0.015
Uniquely regulated by t-TUCB-FLVs				
Anxa5			−0.632	0.034
Arrdc3			−1.218	0.027
Erdr1			1.577	0.025
Gck			−0.662	0.035
Gmppa			0.618	0.027
Rgs16			−1.402	0.039
Rhobtb1			−0.639	0.040
Sult1d1			−0.850	0.035
Txnip			−1.151	0.041
Ugp2			−0.586	0.040

**Table 3:** Gene ontology of EtOH vs. EtOH+t-TUCB-FLVs.

GO:BP Term	GO Term ID	Adj. *p*-Value	Genes
Bile acid biosynthetic process	GO:0006699	0.011	Errfi1, Cyp7b1, Ces1d
Small molecule biosynthetic process	GO:0044283	0.012	Aass, Egr1, Errfi1, Slc39a14, Cyp7b1, Ces1d, Fads2
Circadian rhythm	GO:0007623	0.016	Egr1, Klf10, Npas2, Cyp7b1, Ces1D, Hes6
Organic anion transport	GO:0015711	0.029	Arl6ip5, Slc10a2, Slc39a14, Slc7a2, SlcO1a1, Ces1d
Bile acid metabolic process	GO:0008206	0.042	Errfi1, Cyp7b1, Ces1d

Analyzed using g:Profiler (29).

## Data Availability

The NanoString Digital Spatial Profiling data are available at the Sequence Read Archive (SRA) with the submission ID: PRJNA1308103.

## Supplementary Materials

The following supporting information can be downloaded at: https://www.mdpi.com/article/10.3390/biology14091267/s1, Supplemental Table S1. EtOH vs. PF DEGs.

## Abbreviations

The following abbreviations are used in this manuscript:

ALD	Alcohol-associated liver disease
s-EH	Soluble epoxide hydrolase
EtOH	Ethanol
t-TUCB	trans-4-[4-(3-adamantan-1-yl-ureido)-cyclohexyloxy]-benzoic acid
Ep-FAs	Epoxy fatty acids
Dh-FAs	Dihydroxy fatty acids
PUFAs	Polyunsaturated fatty acids
DOPC	1,2-dioleoyl-sn-glycero-3-phosphocholine
POPA	1-palmitoyl-2-oleoyl-sn-glycero-3-phosphate
FLVs	Fusogenic lipid vesicles
DiD	1,1′-Dioctadecyl-3,3,3′,3′-Tetramethylindodicarbocyanine, 4-Chlorobenzenesulfonate
PF	Pair fed
ALT	Alanine aminotransferase
TUNEL	Terminal deoxynucleotidyl transferase dUTP nick end labeling
CAE	Chloroacetate esterase
TGs	Triglycerides
DSP	Digital Spatial Profiling
AOI	Areas of Interest
WTA	Whole transcriptome analysis
GSEA	Gene Set Enrichment Analysis
GO	Gene Ontology
SEM	Standard error of the mean
ANOVA	Analysis of variance
DEG	Differentially expressed gene
