# Supplementary material for: Hepatocyte-Specific Transcriptional Responses to Liver-Targeted Delivery of a Soluble Epoxide Hydrolase Inhibitor in a Mouse Model of Alcohol-Associated Liver Disease
Source: Biology (Basel). 2025 Sep 13;14(9):1267. doi: 10.3390/biology14091267 (PMC12467949; doi:10.3390/biology14091267)
Supplement: Supplementary file 1 [file biology-14-01267-s001.zip › biology-3825466-supplementary.pdf]

Supplemental Table S1. EtOH vs. PF DEGs

| Gene | log2FC | P value | Adjusted P value | Gene | log2FC | P value | Adjusted P value |
|---|---|---|---|---|---|---|---|
| Mt2 | 7.9548 | 0.0000 | 0.0023 | Emc9 | -0.5915 | 0.0036 | 0.0182 |
| Mt1 | 5.9115 | 0.0001 | 0.0026 | Kynu | -0.5916 | 0.0003 | 0.0053 |
| Lcn2 | 4.8186 | 0.0001 | 0.0032 | Map3k13 | -0.5918 | 0.0037 | 0.0185 |
| Saa2 | 4.7431 | 0.0009 | 0.0087 | Prim2 | -0.5919 | 0.0116 | 0.0370 |
| Cyp2b10 | 4.7318 | 0.0000 | 0.0025 | Plcg2 | -0.5928 | 0.0017 | 0.0121 |
| Saa1 | 4.6793 | 0.0005 | 0.0063 | Smarca2 | -0.5932 | 0.0020 | 0.0131 |
| Trib3 | 3.9442 | 0.0004 | 0.0056 | Pxylp1 | -0.5935 | 0.0067 | 0.0267 |
| Gadd45a | 3.8052 | 0.0091 | 0.0321 | Clec12a | -0.5941 | 0.0040 | 0.0196 |
| Igfbp1 | 3.7914 | 0.0067 | 0.0267 | Tdrd7 | -0.5945 | 0.0053 | 0.0231 |
| Cdkn1a | 3.6434 | 0.0000 | 0.0025 | 4930523C07Rik | -0.5954 | 0.0053 | 0.0231 |
| Egr1 | 3.5531 | 0.0002 | 0.0042 | Rnf145 | -0.5955 | 0.0009 | 0.0089 |
| Insig2 | 3.4048 | 0.0001 | 0.0039 | Sgcd | -0.5958 | 0.0091 | 0.0320 |
| Sgk1 | 3.3824 | 0.0001 | 0.0036 | Gsap | -0.5960 | 0.0031 | 0.0167 |
| Ddit3 | 3.0215 | 0.0007 | 0.0074 | Pex5 | -0.5966 | 0.0008 | 0.0081 |
| LOC118568792 | 3.0200 | 0.0001 | 0.0039 | LOC118567918 | -0.5970 | 0.0056 | 0.0238 |
| Apcs | 2.7811 | 0.0000 | 0.0003 | Kank2 | -0.5973 | 0.0016 | 0.0120 |
| Serpina3n | 2.7430 | 0.0012 | 0.0101 | Psmb3 | -0.5975 | 0.0000 | 0.0015 |
| Chka | 2.6928 | 0.0003 | 0.0049 | Acox3 | -0.5976 | 0.0002 | 0.0041 |
| Atf3 | 2.6145 | 0.0041 | 0.0198 | Adi1 | -0.5983 | 0.0003 | 0.0054 |
| Ctsl | 2.5953 | 0.0000 | 0.0016 | Suv39h1 | -0.5988 | 0.0036 | 0.0181 |
| Gpat3 | 2.5591 | 0.0012 | 0.0099 | Rnf152 | -0.5994 | 0.0007 | 0.0075 |
| Fgl1 | 2.5475 | 0.0001 | 0.0039 | Mgam | -0.5996 | 0.0016 | 0.0118 |
| Serpina3m | 2.4530 | 0.0002 | 0.0042 | A1cf | -0.5998 | 0.0007 | 0.0076 |
| Atf5 | 2.4406 | 0.0003 | 0.0050 | Camk2n1 | -0.6003 | 0.0036 | 0.0184 |
| E030018B13Rik | 2.3978 | 0.0000 | 0.0010 | Rps6ka1 | -0.6003 | 0.0128 | 0.0395 |
| LOC118568705 | 2.3495 | 0.0012 | 0.0102 | Pla2g2d | -0.6005 | 0.0125 | 0.0391 |
| Acnat2 | 2.3037 | 0.0014 | 0.0110 | Mlec | -0.6008 | 0.0029 | 0.0161 |
| Trp53inp1 | 2.2788 | 0.0064 | 0.0259 | Pim1 | -0.6017 | 0.0017 | 0.0121 |
| Plin2 | 2.2709 | 0.0002 | 0.0040 | Glo1 | -0.6018 | 0.0018 | 0.0126 |
| Fkbp5 | 2.2261 | 0.0001 | 0.0028 | Rassf3 | -0.6018 | 0.0012 | 0.0102 |
| Cidec | 2.2056 | 0.0001 | 0.0033 | Spout1 | -0.6024 | 0.0010 | 0.0092 |
| Ddit4 | 2.1976 | 0.0031 | 0.0167 | Cog4 | -0.6032 | 0.0001 | 0.0030 |
| Sqstm1 | 2.1839 | 0.0001 | 0.0033 | Cndp2 | -0.6039 | 0.0004 | 0.0060 |
| Gdf15 | 2.1591 | 0.0006 | 0.0068 | Pah | -0.6042 | 0.0019 | 0.0129 |
| Leap2 | 2.1582 | 0.0004 | 0.0054 | Lrrc3 | -0.6044 | 0.0014 | 0.0110 |
| Fga | 2.1253 | 0.0011 | 0.0096 | Mrc1 | -0.6048 | 0.0047 | 0.0217 |
| H3f3b | 2.1145 | 0.0003 | 0.0051 | Svip | -0.6056 | 0.0021 | 0.0134 |
| Rora | 2.1095 | 0.0006 | 0.0073 | 4930451I11Rik | -0.6060 | 0.0120 | 0.0379 |
| Actg1 | 2.0994 | 0.0005 | 0.0064 | Aard | -0.6067 | 0.0169 | 0.0471 |

| | | | | | | | |
|---|---|---|---|---|---|---|---|
| Aass | 2.0858 | 0.0000 | 0.0014 | Cox6b1 | -0.6068 | 0.0002 | 0.0042 |
| Fgg | 2.0588 | 0.0006 | 0.0070 | Phc1 | -0.6068 | 0.0087 | 0.0312 |
| Sult1a1 | 2.0536 | 0.0000 | 0.0000 | Ndst1 | -0.6069 | 0.0061 | 0.0253 |
| Ptp4a1 | 2.0412 | 0.0005 | 0.0067 | Prkra | -0.6071 | 0.0141 | 0.0421 |
| Lrg1 | 2.0350 | 0.0006 | 0.0070 | Sptbn2 | -0.6083 | 0.0012 | 0.0100 |
| Mideas | 2.0190 | 0.0029 | 0.0162 | P2ry4 | -0.6083 | 0.0010 | 0.0094 |
| Angptl4 | 1.9994 | 0.0002 | 0.0040 | 0610012G03Rik | -0.6085 | 0.0007 | 0.0075 |
| Gpt2 | 1.9820 | 0.0000 | 0.0015 | Ago1 | -0.6088 | 0.0029 | 0.0163 |
| St3gal5 | 1.9806 | 0.0000 | 0.0015 | Clec4g | -0.6088 | 0.0047 | 0.0216 |
| Hspa5 | 1.9745 | 0.0015 | 0.0112 | Cyp3a25 | -0.6093 | 0.0034 | 0.0176 |
| Nnmt | 1.9395 | 0.0005 | 0.0062 | Abca16 | -0.6095 | 0.0121 | 0.0382 |
| Soat2 | 1.9314 | 0.0003 | 0.0049 | Cpne2 | -0.6097 | 0.0011 | 0.0095 |
| Rbpms | 1.9223 | 0.0001 | 0.0029 | Abcg4 | -0.6109 | 0.0052 | 0.0229 |
| Il1r1 | 1.8967 | 0.0000 | 0.0015 | Tnfaip8l1 | -0.6115 | 0.0019 | 0.0129 |
| Serpina7 | 1.8926 | 0.0142 | 0.0423 | 4930564C03Rik | -0.6116 | 0.0081 | 0.0297 |
| Cyp4a14 | 1.8423 | 0.0002 | 0.0048 | Eci2 | -0.6118 | 0.0008 | 0.0083 |
| Hp | 1.8385 | 0.0000 | 0.0022 | Zfp36 | -0.6127 | 0.0004 | 0.0056 |
| Tnfrsf12a | 1.8297 | 0.0011 | 0.0098 | Pdk1 | -0.6132 | 0.0011 | 0.0098 |
| Stra6l | 1.8249 | 0.0000 | 0.0015 | Rab3a | -0.6133 | 0.0043 | 0.0203 |
| Lbp | 1.8183 | 0.0000 | 0.0015 | Dclre1c | -0.6134 | 0.0009 | 0.0086 |
| Fgb | 1.8052 | 0.0008 | 0.0083 | Elof1 | -0.6139 | 0.0025 | 0.0148 |
| Cyb5r1 | 1.7781 | 0.0000 | 0.0025 | Tmprss6 | -0.6139 | 0.0054 | 0.0233 |
| Cyp3a13 | 1.7741 | 0.0002 | 0.0042 | Dym | -0.6142 | 0.0022 | 0.0140 |
| Tsku | 1.7737 | 0.0007 | 0.0077 | Marf1 | -0.6152 | 0.0004 | 0.0058 |
| Orm2 | 1.7588 | 0.0000 | 0.0016 | Cd74 | -0.6152 | 0.0046 | 0.0214 |
| Tgm2 | 1.7525 | 0.0000 | 0.0015 | Isoc2a | -0.6152 | 0.0003 | 0.0050 |
| Ddx28 | 1.7471 | 0.0001 | 0.0036 | Izumo4 | -0.6159 | 0.0001 | 0.0028 |
| Pid1 | 1.7422 | 0.0001 | 0.0036 | Cacybp | -0.6165 | 0.0005 | 0.0068 |
| Fgf21 | 1.7088 | 0.0133 | 0.0407 | Abcg3 | -0.6168 | 0.0037 | 0.0185 |
| Asns | 1.7057 | 0.0009 | 0.0087 | Pmf1 | -0.6176 | 0.0015 | 0.0116 |
| Hmox1 | 1.7003 | 0.0077 | 0.0288 | Grem2 | -0.6188 | 0.0006 | 0.0071 |
| Cln8 | 1.6909 | 0.0008 | 0.0083 | Cdk5 | -0.6194 | 0.0003 | 0.0048 |
| Nfil3 | 1.6897 | 0.0001 | 0.0037 | Ctag2l1 | -0.6198 | 0.0027 | 0.0156 |
| Btg2 | 1.6638 | 0.0041 | 0.0198 | Rnf186 | -0.6199 | 0.0074 | 0.0282 |
| Irf2bp2 | 1.6608 | 0.0009 | 0.0089 | Dbil5 | -0.6205 | 0.0044 | 0.0208 |
| Orm1 | 1.6583 | 0.0013 | 0.0103 | Tsen34 | -0.6213 | 0.0051 | 0.0227 |
| Slc39a14 | 1.6406 | 0.0000 | 0.0015 | Slc40a1 | -0.6215 | 0.0002 | 0.0045 |
| Clpx | 1.6076 | 0.0001 | 0.0028 | Ptprc | -0.6215 | 0.0118 | 0.0375 |
| Gtpbp2 | 1.5857 | 0.0004 | 0.0055 | Dnpep | -0.6216 | 0.0002 | 0.0042 |
| Mat1a | 1.5618 | 0.0000 | 0.0015 | Dcps | -0.6216 | 0.0028 | 0.0159 |
| Gnpnat1 | 1.5427 | 0.0010 | 0.0091 | Klf13 | -0.6218 | 0.0002 | 0.0039 |

| | | | | | | | |
|---|---|---|---|---|---|---|---|
| Retreg1 | 1.5309 | 0.0008 | 0.0081 | Gins4 | -0.6222 | 0.0056 | 0.0239 |
| Ormdl3 | 1.5264 | 0.0008 | 0.0081 | H2bu2 | -0.6223 | 0.0053 | 0.0232 |
| Slc25a22 | 1.5242 | 0.0001 | 0.0028 | Chp2 | -0.6228 | 0.0161 | 0.0458 |
| Klf6 | 1.5213 | 0.0041 | 0.0197 | Hsph1 | -0.6233 | 0.0005 | 0.0066 |
| Itih2 | 1.5047 | 0.0000 | 0.0013 | Cotl1 | -0.6242 | 0.0049 | 0.0222 |
| Slc16a5 | 1.4983 | 0.0004 | 0.0060 | Ttr | -0.6243 | 0.0055 | 0.0236 |
| Cdo1 | 1.4793 | 0.0000 | 0.0013 | Otc | -0.6250 | 0.0091 | 0.0320 |
| Abhd2 | 1.4724 | 0.0000 | 0.0010 | Tpcn1 | -0.6254 | 0.0002 | 0.0042 |
| S100a10 | 1.4685 | 0.0000 | 0.0021 | Gstk1 | -0.6256 | 0.0017 | 0.0122 |
| Myc | 1.4652 | 0.0037 | 0.0185 | Rac2 | -0.6258 | 0.0051 | 0.0226 |
| Acox2 | 1.4650 | 0.0000 | 0.0015 | Csf1r | -0.6266 | 0.0001 | 0.0028 |
| Sdsl | 1.4587 | 0.0044 | 0.0208 | Rbfa | -0.6268 | 0.0012 | 0.0100 |
| Arl4a | 1.4523 | 0.0072 | 0.0279 | B2m | -0.6271 | 0.0049 | 0.0221 |
| Gstt3 | 1.4435 | 0.0002 | 0.0042 | Mtfp1 | -0.6281 | 0.0008 | 0.0085 |
| Htatip2 | 1.4431 | 0.0008 | 0.0085 | 4933407O12Rik | -0.6282 | 0.0089 | 0.0316 |
| Ppp1r15a | 1.4392 | 0.0002 | 0.0045 | Rilp | -0.6285 | 0.0052 | 0.0229 |
| Itih3 | 1.4375 | 0.0042 | 0.0200 | Ola1 | -0.6287 | 0.0003 | 0.0051 |
| Tacc2 | 1.4312 | 0.0000 | 0.0015 | Pcyox1 | -0.6288 | 0.0003 | 0.0052 |
| Npc1 | 1.4243 | 0.0001 | 0.0036 | Ighm | -0.6289 | 0.0124 | 0.0389 |
| Cyp2a22 | 1.4080 | 0.0002 | 0.0042 | Pla2g4d | -0.6290 | 0.0186 | 0.0499 |
| Prg4 | 1.4021 | 0.0001 | 0.0037 | Rab3b | -0.6301 | 0.0072 | 0.0279 |
| Dpp9 | 1.3996 | 0.0001 | 0.0026 | Sesn3 | -0.6301 | 0.0004 | 0.0058 |
| Nlrp12 | 1.3988 | 0.0001 | 0.0039 | Pex26 | -0.6303 | 0.0008 | 0.0083 |
| Fibin | 1.3800 | 0.0000 | 0.0020 | Ugdh | -0.6315 | 0.0030 | 0.0163 |
| Kyat1 | 1.3742 | 0.0001 | 0.0037 | Prkd3 | -0.6318 | 0.0003 | 0.0054 |
| Slc25a51 | 1.3585 | 0.0001 | 0.0039 | Rmnd1 | -0.6320 | 0.0000 | 0.0022 |
| Rab30 | 1.3530 | 0.0001 | 0.0031 | Tmem131l | -0.6321 | 0.0036 | 0.0181 |
| Cyp26b1 | 1.3469 | 0.0019 | 0.0128 | Abtb1 | -0.6328 | 0.0006 | 0.0073 |
| Bhmt | 1.3435 | 0.0020 | 0.0133 | 9330182O14Rik | -0.6332 | 0.0021 | 0.0134 |
| Sat1 | 1.3370 | 0.0004 | 0.0060 | Echdc1 | -0.6333 | 0.0000 | 0.0017 |
| Hsp90b1 | 1.3366 | 0.0019 | 0.0130 | Ccdc57 | -0.6340 | 0.0003 | 0.0052 |
| G0s2 | 1.3344 | 0.0009 | 0.0089 | Samhd1 | -0.6343 | 0.0049 | 0.0221 |
| Cpeb4 | 1.3287 | 0.0000 | 0.0018 | Nipsnap1 | -0.6349 | 0.0032 | 0.0172 |
| Rarres1 | 1.3245 | 0.0000 | 0.0015 | Dhtkd1 | -0.6351 | 0.0058 | 0.0244 |
| Ets2 | 1.3177 | 0.0059 | 0.0245 | Fdxr | -0.6355 | 0.0004 | 0.0057 |
| Steap4 | 1.3130 | 0.0012 | 0.0100 | Ssbp3 | -0.6358 | 0.0013 | 0.0103 |
| Epb41 | 1.3035 | 0.0000 | 0.0014 | Olfr1033 | -0.6359 | 0.0064 | 0.0261 |
| Cxcl1 | 1.3006 | 0.0011 | 0.0097 | Acad8 | -0.6361 | 0.0006 | 0.0070 |
| Dusp6 | 1.2864 | 0.0024 | 0.0146 | Cyp2r1 | -0.6367 | 0.0003 | 0.0050 |
| Nop58 | 1.2847 | 0.0001 | 0.0028 | Col5a2 | -0.6370 | 0.0076 | 0.0286 |
| Gfpt1 | 1.2839 | 0.0000 | 0.0025 | Aldh1l1 | -0.6386 | 0.0092 | 0.0322 |

| | | | | | | | |
|---|---|---|---|---|---|---|---|
| Cyp4a10 | 1.2814 | 0.0014 | 0.0111 | A930033H14Rik | -0.6388 | 0.0027 | 0.0154 |
| Rnf125 | 1.2806 | 0.0009 | 0.0089 | Ephx2 | -0.6391 | 0.0000 | 0.0019 |
| Golim4 | 1.2773 | 0.0000 | 0.0013 | Hikeshi | -0.6412 | 0.0172 | 0.0475 |
| Ccdc47 | 1.2750 | 0.0004 | 0.0060 | Idi1 | -0.6417 | 0.0022 | 0.0140 |
| Gars | 1.2722 | 0.0000 | 0.0017 | Serhl | -0.6421 | 0.0003 | 0.0051 |
| Snx10 | 1.2679 | 0.0000 | 0.0015 | Nsdhl | -0.6422 | 0.0012 | 0.0100 |
| Acsl4 | 1.2648 | 0.0033 | 0.0173 | Cd22 | -0.6426 | 0.0168 | 0.0470 |
| Grn | 1.2603 | 0.0000 | 0.0015 | Acaca | -0.6432 | 0.0027 | 0.0156 |
| Foxa3 | 1.2592 | 0.0008 | 0.0081 | Ropn1l | -0.6432 | 0.0022 | 0.0138 |
| Taf15 | 1.2566 | 0.0000 | 0.0015 | Dab2 | -0.6435 | 0.0115 | 0.0370 |
| Gjb2 | 1.2532 | 0.0004 | 0.0058 | Klhl21 | -0.6437 | 0.0004 | 0.0056 |
| Avpr1a | 1.2506 | 0.0032 | 0.0170 | Pbld1 | -0.6450 | 0.0001 | 0.0032 |
| Pctp | 1.2473 | 0.0000 | 0.0015 | Csrnp1 | -0.6456 | 0.0008 | 0.0081 |
| Klf9 | 1.2401 | 0.0004 | 0.0056 | Lgr4 | -0.6463 | 0.0012 | 0.0100 |
| Tmem62 | 1.2381 | 0.0000 | 0.0017 | Fbxo9 | -0.6466 | 0.0020 | 0.0131 |
| Fst | 1.2280 | 0.0165 | 0.0464 | Gpr146 | -0.6475 | 0.0033 | 0.0172 |
| Pla2g12a | 1.2267 | 0.0014 | 0.0110 | 1810046K07Rik | -0.6482 | 0.0132 | 0.0405 |
| Arsg | 1.2180 | 0.0000 | 0.0015 | Abcd2 | -0.6482 | 0.0016 | 0.0120 |
| Sco2 | 1.2112 | 0.0004 | 0.0055 | Erg28 | -0.6492 | 0.0001 | 0.0035 |
| Stbd1 | 1.2054 | 0.0043 | 0.0204 | Palld | -0.6493 | 0.0030 | 0.0165 |
| Litaf | 1.1937 | 0.0000 | 0.0024 | Ugt1a5 | -0.6494 | 0.0010 | 0.0094 |
| Slc16a6 | 1.1763 | 0.0002 | 0.0042 | Aldh1a1 | -0.6511 | 0.0152 | 0.0443 |
| Arntl | 1.1746 | 0.0002 | 0.0048 | Dhrs1 | -0.6513 | 0.0010 | 0.0092 |
| Pnrc2 | 1.1726 | 0.0001 | 0.0029 | Rpain | -0.6519 | 0.0026 | 0.0152 |
| Dop1b | 1.1699 | 0.0004 | 0.0060 | Epb41l4b | -0.6536 | 0.0040 | 0.0195 |
| Klf10 | 1.1694 | 0.0001 | 0.0035 | Cyp2d12 | -0.6539 | 0.0001 | 0.0033 |
| Pxmp4 | 1.1637 | 0.0003 | 0.0048 | Haus4 | -0.6546 | 0.0063 | 0.0258 |
| Slc25a47 | 1.1621 | 0.0004 | 0.0058 | Ndufb3 | -0.6550 | 0.0005 | 0.0063 |
| Sdc4 | 1.1608 | 0.0017 | 0.0121 | Cxcr3 | -0.6563 | 0.0178 | 0.0484 |
| Tat | 1.1603 | 0.0006 | 0.0071 | Selenoo | -0.6565 | 0.0000 | 0.0010 |
| Itih4 | 1.1567 | 0.0020 | 0.0132 | 4930578L24Rik | -0.6565 | 0.0113 | 0.0365 |
| Rgs7bp | 1.1544 | 0.0002 | 0.0041 | Sbk1 | -0.6576 | 0.0002 | 0.0041 |
| Ifrd1 | 1.1495 | 0.0081 | 0.0297 | Kit | -0.6603 | 0.0058 | 0.0242 |
| Fbxo6 | 1.1470 | 0.0001 | 0.0029 | Tyrobp | -0.6632 | 0.0051 | 0.0226 |
| Hamp | 1.1456 | 0.0005 | 0.0067 | Sult1c2 | -0.6633 | 0.0021 | 0.0135 |
| Lpgat1 | 1.1326 | 0.0003 | 0.0052 | Dmac2 | -0.6636 | 0.0018 | 0.0125 |
| Rnf149 | 1.1313 | 0.0001 | 0.0037 | Gm46272 | -0.6639 | 0.0003 | 0.0049 |
| Slc3a2 | 1.1299 | 0.0001 | 0.0032 | Nlrp6 | -0.6640 | 0.0013 | 0.0105 |
| Mknk2 | 1.1281 | 0.0005 | 0.0068 | Nr1h5 | -0.6648 | 0.0163 | 0.0460 |
| Myadm | 1.1264 | 0.0000 | 0.0021 | Bri3 | -0.6654 | 0.0029 | 0.0161 |
| Slc4a4 | 1.1158 | 0.0000 | 0.0013 | Folr2 | -0.6655 | 0.0042 | 0.0202 |

| | | | | | | | |
|---|---|---|---|---|---|---|---|
| Btg1 | 1.1040 | 0.0004 | 0.0055 | Cflar | -0.6663 | 0.0004 | 0.0055 |
| Tbl1xr1 | 1.1018 | 0.0009 | 0.0089 | Stk16 | -0.6663 | 0.0032 | 0.0171 |
| Tm4sf4 | 1.0996 | 0.0001 | 0.0026 | Prdx6 | -0.6671 | 0.0026 | 0.0153 |
| Col4a1 | 1.0945 | 0.0092 | 0.0322 | Ctrc | -0.6672 | 0.0154 | 0.0445 |
| Sgk2 | 1.0925 | 0.0001 | 0.0033 | 1110002E22Rik | -0.6673 | 0.0087 | 0.0311 |
| Hjv | 1.0920 | 0.0001 | 0.0030 | Proz | -0.6685 | 0.0015 | 0.0115 |
| Junb | 1.0821 | 0.0001 | 0.0032 | Ces2c | -0.6692 | 0.0155 | 0.0448 |
| Cbx4 | 1.0812 | 0.0003 | 0.0052 | Ctc1 | -0.6693 | 0.0031 | 0.0166 |
| Ralgapa2 | 1.0810 | 0.0000 | 0.0023 | Lrp4 | -0.6702 | 0.0012 | 0.0100 |
| Il1rn | 1.0785 | 0.0119 | 0.0378 | Fam210a | -0.6704 | 0.0009 | 0.0090 |
| Epas1 | 1.0772 | 0.0001 | 0.0031 | Abcd1 | -0.6753 | 0.0001 | 0.0029 |
| Coq8b | 1.0761 | 0.0000 | 0.0013 | Rsrp1 | -0.6753 | 0.0004 | 0.0055 |
| C3 | 1.0746 | 0.0008 | 0.0081 | Rnaseh2c | -0.6755 | 0.0000 | 0.0010 |
| Crcp | 1.0742 | 0.0000 | 0.0017 | Slc47a1 | -0.6775 | 0.0022 | 0.0139 |
| Plin5 | 1.0731 | 0.0015 | 0.0112 | Mme | -0.6803 | 0.0015 | 0.0116 |
| Shfl | 1.0710 | 0.0000 | 0.0013 | Slc22a1 | -0.6809 | 0.0146 | 0.0431 |
| Ube2v2 | 1.0708 | 0.0003 | 0.0050 | Rmdn2 | -0.6819 | 0.0005 | 0.0063 |
| H1f2 | 1.0683 | 0.0020 | 0.0131 | Aldh7a1 | -0.6819 | 0.0013 | 0.0106 |
| Hmox2 | 1.0675 | 0.0000 | 0.0011 | Lcp1 | -0.6830 | 0.0003 | 0.0051 |
| Eif4g3 | 1.0672 | 0.0005 | 0.0061 | Pepd | -0.6836 | 0.0002 | 0.0042 |
| Picalm | 1.0664 | 0.0003 | 0.0049 | Rhof | -0.6848 | 0.0014 | 0.0112 |
| Atp2a2 | 1.0621 | 0.0000 | 0.0017 | Pck1 | -0.6867 | 0.0034 | 0.0177 |
| Gm3893 | 1.0602 | 0.0005 | 0.0067 | S1pr1 | -0.6869 | 0.0002 | 0.0045 |
| Ube2g2 | 1.0571 | 0.0006 | 0.0071 | Map2k3 | -0.6870 | 0.0027 | 0.0154 |
| Jund | 1.0545 | 0.0045 | 0.0209 | Dhdh | -0.6878 | 0.0001 | 0.0031 |
| Serpina10 | 1.0525 | 0.0005 | 0.0067 | Gas1 | -0.6878 | 0.0023 | 0.0143 |
| Slc25a15 | 1.0496 | 0.0000 | 0.0017 | Hdac11 | -0.6889 | 0.0005 | 0.0061 |
| Ccl9 | 1.0482 | 0.0011 | 0.0095 | Rara | -0.6898 | 0.0088 | 0.0314 |
| Slc41a2 | 1.0476 | 0.0009 | 0.0088 | Gpr155 | -0.6900 | 0.0013 | 0.0106 |
| Btg3 | 1.0407 | 0.0027 | 0.0156 | Apoc2 | -0.6903 | 0.0050 | 0.0224 |
| Jun | 1.0403 | 0.0083 | 0.0300 | Slc6a6 | -0.6921 | 0.0020 | 0.0131 |
| Gm3776 | 1.0389 | 0.0002 | 0.0045 | Cyb5b | -0.6921 | 0.0037 | 0.0186 |
| Creld2 | 1.0373 | 0.0077 | 0.0289 | Abat | -0.6923 | 0.0031 | 0.0168 |
| Cyp17a1 | 1.0336 | 0.0081 | 0.0297 | Fnip2 | -0.6929 | 0.0002 | 0.0042 |
| Krt18 | 1.0325 | 0.0003 | 0.0053 | Ugt2a3 | -0.6931 | 0.0040 | 0.0194 |
| Acot4 | 1.0321 | 0.0010 | 0.0093 | Nelfe | -0.6931 | 0.0003 | 0.0053 |
| Sdr42e1 | 1.0288 | 0.0065 | 0.0263 | Fam20c | -0.6937 | 0.0008 | 0.0081 |
| Pnp | 1.0278 | 0.0001 | 0.0031 | 4930595D18Rik | -0.6943 | 0.0122 | 0.0384 |
| Phospho1 | 1.0210 | 0.0015 | 0.0113 | Rbm47 | -0.6943 | 0.0000 | 0.0010 |
| Mcfd2 | 1.0158 | 0.0000 | 0.0017 | Klf12 | -0.6965 | 0.0007 | 0.0078 |
| Por | 1.0127 | 0.0004 | 0.0057 | Cct8 | -0.6974 | 0.0003 | 0.0048 |

| | | | | | | | |
|---|---|---|---|---|---|---|---|
| Dennd4a | 1.0114 | 0.0001 | 0.0028 | Paox | -0.6980 | 0.0006 | 0.0071 |
| Hpx | 1.0100 | 0.0019 | 0.0130 | Cd200r3 | -0.6986 | 0.0153 | 0.0443 |
| H6pd | 1.0077 | 0.0000 | 0.0022 | F8 | -0.6995 | 0.0052 | 0.0229 |
| Rnf185 | 1.0066 | 0.0006 | 0.0070 | Fgfr4 | -0.6998 | 0.0026 | 0.0153 |
| Smox | 1.0064 | 0.0080 | 0.0294 | Pkd1l3 | -0.7009 | 0.0055 | 0.0236 |
| F11r | 1.0028 | 0.0000 | 0.0017 | Vwce | -0.7013 | 0.0000 | 0.0015 |
| Myh9 | 0.9973 | 0.0000 | 0.0013 | Bbox1 | -0.7017 | 0.0001 | 0.0028 |
| Slc35g1 | 0.9937 | 0.0008 | 0.0082 | Lrp1 | -0.7021 | 0.0068 | 0.0268 |
| Mrap | 0.9920 | 0.0003 | 0.0052 | Nelfcd | -0.7031 | 0.0004 | 0.0056 |
| Qsox1 | 0.9917 | 0.0009 | 0.0088 | Apof | -0.7044 | 0.0050 | 0.0223 |
| Fyb2 | 0.9901 | 0.0018 | 0.0126 | Csk | -0.7052 | 0.0004 | 0.0054 |
| Galnt2 | 0.9839 | 0.0001 | 0.0032 | Cish | -0.7061 | 0.0008 | 0.0086 |
| Cd36 | 0.9831 | 0.0069 | 0.0272 | Mdh1 | -0.7064 | 0.0013 | 0.0105 |
| Ahsg | 0.9823 | 0.0008 | 0.0081 | Txn1 | -0.7065 | 0.0001 | 0.0031 |
| Lgalsl | 0.9818 | 0.0011 | 0.0095 | Aadac | -0.7083 | 0.0000 | 0.0014 |
| Arl6ip5 | 0.9806 | 0.0001 | 0.0037 | Msh3 | -0.7102 | 0.0000 | 0.0017 |
| Dstn | 0.9763 | 0.0001 | 0.0036 | Dclk3 | -0.7130 | 0.0078 | 0.0290 |
| Rchy1 | 0.9673 | 0.0000 | 0.0021 | Gpd1l | -0.7134 | 0.0002 | 0.0045 |
| Slc41a3 | 0.9660 | 0.0000 | 0.0015 | Pfkm | -0.7136 | 0.0053 | 0.0230 |
| Sqle | 0.9646 | 0.0002 | 0.0045 | Clcn2 | -0.7148 | 0.0037 | 0.0185 |
| Inhbb | 0.9628 | 0.0015 | 0.0113 | Shank2 | -0.7149 | 0.0002 | 0.0045 |
| Lepr | 0.9621 | 0.0023 | 0.0143 | Gcdh | -0.7175 | 0.0008 | 0.0081 |
| Cald1 | 0.9619 | 0.0001 | 0.0031 | 3110067G11Rik | -0.7199 | 0.0146 | 0.0432 |
| Gdap2 | 0.9555 | 0.0010 | 0.0094 | Scart1 | -0.7203 | 0.0066 | 0.0264 |
| Orm3 | 0.9537 | 0.0017 | 0.0122 | Slc25a11 | -0.7205 | 0.0002 | 0.0041 |
| Sesn2 | 0.9530 | 0.0021 | 0.0134 | Rdh11 | -0.7244 | 0.0002 | 0.0045 |
| Ptbp1 | 0.9530 | 0.0004 | 0.0054 | Cmah | -0.7258 | 0.0092 | 0.0323 |
| Srxn1 | 0.9522 | 0.0001 | 0.0035 | Lactb | -0.7266 | 0.0013 | 0.0107 |
| Nedd4l | 0.9478 | 0.0006 | 0.0072 | Zfpm1 | -0.7270 | 0.0005 | 0.0061 |
| Sec22b | 0.9458 | 0.0000 | 0.0010 | Chac2 | -0.7278 | 0.0009 | 0.0089 |
| Dusp16 | 0.9396 | 0.0069 | 0.0272 | Haao | -0.7289 | 0.0021 | 0.0136 |
| Extl1 | 0.9285 | 0.0000 | 0.0022 | Fahd1 | -0.7295 | 0.0001 | 0.0033 |
| Arhgap5 | 0.9261 | 0.0001 | 0.0028 | Mov10 | -0.7296 | 0.0020 | 0.0131 |
| Plpp5 | 0.9215 | 0.0003 | 0.0049 | Cpped1 | -0.7297 | 0.0005 | 0.0065 |
| Marchf6 | 0.9203 | 0.0000 | 0.0015 | Lrit2 | -0.7322 | 0.0001 | 0.0028 |
| Zbtb16 | 0.9190 | 0.0001 | 0.0035 | Fchsd2 | -0.7337 | 0.0001 | 0.0038 |
| Baiap2l1 | 0.9158 | 0.0026 | 0.0151 | Ugt1a6b | -0.7348 | 0.0001 | 0.0032 |
| Abhd17c | 0.9144 | 0.0000 | 0.0017 | Aqp11 | -0.7349 | 0.0016 | 0.0119 |
| Arhgef2 | 0.9121 | 0.0144 | 0.0428 | Idh1 | -0.7365 | 0.0001 | 0.0037 |
| Ddc | 0.9116 | 0.0000 | 0.0025 | Arhgef19 | -0.7382 | 0.0008 | 0.0085 |
| Got1 | 0.9087 | 0.0007 | 0.0076 | Rbbp4 | -0.7391 | 0.0009 | 0.0089 |

| | | | | | | | |
|---|---|---|---|---|---|---|---|
| Nrbp2 | 0.9076 | 0.0000 | 0.0014 | Actn1 | -0.7399 | 0.0022 | 0.0140 |
| B3galt1 | 0.9074 | 0.0087 | 0.0311 | Cfap20 | -0.7403 | 0.0001 | 0.0036 |
| Tab2 | 0.9067 | 0.0003 | 0.0049 | Cd5l | -0.7428 | 0.0026 | 0.0151 |
| Lrrc8a | 0.9049 | 0.0001 | 0.0037 | Afm | -0.7433 | 0.0001 | 0.0037 |
| Aebp2 | 0.9030 | 0.0002 | 0.0042 | Ugt2b36 | -0.7454 | 0.0004 | 0.0056 |
| Fh1 | 0.9027 | 0.0006 | 0.0070 | Abca2 | -0.7456 | 0.0006 | 0.0068 |
| Slc25a33 | 0.9019 | 0.0004 | 0.0060 | Stard7 | -0.7458 | 0.0042 | 0.0200 |
| Blcap | 0.9013 | 0.0000 | 0.0017 | Ces1d | -0.7459 | 0.0001 | 0.0035 |
| Ppp1r3b | 0.9004 | 0.0000 | 0.0018 | Nme7 | -0.7475 | 0.0055 | 0.0235 |
| Sdc1 | 0.8982 | 0.0003 | 0.0053 | Cyp2j9 | -0.7478 | 0.0030 | 0.0163 |
| Tmem87b | 0.8859 | 0.0002 | 0.0042 | Abcg2 | -0.7478 | 0.0008 | 0.0085 |
| Hspb8 | 0.8843 | 0.0031 | 0.0167 | Ccnd1 | -0.7487 | 0.0073 | 0.0280 |
| Chchd10 | 0.8825 | 0.0006 | 0.0069 | Zfp395 | -0.7500 | 0.0012 | 0.0102 |
| Ier5 | 0.8785 | 0.0001 | 0.0029 | Coq10b | -0.7505 | 0.0024 | 0.0146 |
| Mmadhc | 0.8785 | 0.0005 | 0.0062 | Nr0b2 | -0.7507 | 0.0048 | 0.0218 |
| Atf6 | 0.8755 | 0.0000 | 0.0013 | Nqo2 | -0.7520 | 0.0010 | 0.0090 |
| Derl1 | 0.8735 | 0.0008 | 0.0081 | Ngef | -0.7520 | 0.0043 | 0.0203 |
| Lrrfip1 | 0.8731 | 0.0002 | 0.0045 | Tedc2 | -0.7523 | 0.0040 | 0.0195 |
| Ppl | 0.8656 | 0.0007 | 0.0075 | Pcsk9 | -0.7536 | 0.0032 | 0.0170 |
| Cxadr | 0.8654 | 0.0043 | 0.0204 | Aspg | -0.7550 | 0.0029 | 0.0161 |
| Dnajc3 | 0.8627 | 0.0002 | 0.0043 | Cyp4f14 | -0.7553 | 0.0009 | 0.0089 |
| Denn2b | 0.8625 | 0.0012 | 0.0100 | Sema4g | -0.7567 | 0.0074 | 0.0282 |
| Eif6 | 0.8619 | 0.0000 | 0.0013 | Cd180 | -0.7587 | 0.0174 | 0.0479 |
| Slc16a1 | 0.8583 | 0.0006 | 0.0070 | Chdh | -0.7591 | 0.0000 | 0.0021 |
| Klf3 | 0.8574 | 0.0172 | 0.0475 | Per3 | -0.7641 | 0.0008 | 0.0086 |
| Gfra1 | 0.8519 | 0.0002 | 0.0045 | Fbxo3 | -0.7653 | 0.0000 | 0.0020 |
| Sall1 | 0.8500 | 0.0016 | 0.0117 | Brap | -0.7657 | 0.0026 | 0.0151 |
| Ern1 | 0.8479 | 0.0010 | 0.0095 | Noct | -0.7673 | 0.0045 | 0.0211 |
| N4bp2l1 | 0.8474 | 0.0052 | 0.0229 | Atl3 | -0.7690 | 0.0003 | 0.0052 |
| Actb | 0.8389 | 0.0025 | 0.0150 | Ciita | -0.7691 | 0.0115 | 0.0368 |
| Sorbs2 | 0.8369 | 0.0159 | 0.0456 | Dglucy | -0.7698 | 0.0000 | 0.0020 |
| Fabp4 | 0.8369 | 0.0102 | 0.0344 | Arid5b | -0.7713 | 0.0003 | 0.0052 |
| Eif3a | 0.8363 | 0.0000 | 0.0015 | Inhbc | -0.7750 | 0.0028 | 0.0158 |
| Krt8 | 0.8329 | 0.0004 | 0.0054 | Apol9a | -0.7766 | 0.0006 | 0.0068 |
| Cry1 | 0.8298 | 0.0002 | 0.0042 | Foxo3 | -0.7771 | 0.0000 | 0.0017 |
| Ccnl1 | 0.8280 | 0.0012 | 0.0101 | Olfm3 | -0.7803 | 0.0015 | 0.0113 |
| Ier2 | 0.8265 | 0.0003 | 0.0052 | Paqr7 | -0.7811 | 0.0011 | 0.0095 |
| Inhbe | 0.8263 | 0.0024 | 0.0147 | Pqbp1 | -0.7816 | 0.0004 | 0.0058 |
| Atp6v0e | 0.8249 | 0.0000 | 0.0025 | Olfml1 | -0.7818 | 0.0004 | 0.0061 |
| Odc1 | 0.8216 | 0.0002 | 0.0045 | Carmil1 | -0.7822 | 0.0002 | 0.0042 |
| Nudt4 | 0.8211 | 0.0001 | 0.0037 | Miga2 | -0.7829 | 0.0001 | 0.0039 |

| | | | | | | | |
|---|---|---|---|---|---|---|---|
| Map3k5 | 0.8203 | 0.0126 | 0.0393 | Rangrf | -0.7869 | 0.0011 | 0.0097 |
| Hyou1 | 0.8185 | 0.0059 | 0.0246 | Akr1c13 | -0.7872 | 0.0006 | 0.0069 |
| Lyve1 | 0.8155 | 0.0034 | 0.0177 | Pipox | -0.7884 | 0.0000 | 0.0025 |
| Plscr1 | 0.8146 | 0.0075 | 0.0283 | Msmo1 | -0.7897 | 0.0023 | 0.0144 |
| Mgst3 | 0.8135 | 0.0030 | 0.0163 | Gk | -0.7908 | 0.0001 | 0.0035 |
| Tpm1 | 0.8108 | 0.0001 | 0.0028 | H2az2 | -0.7913 | 0.0000 | 0.0025 |
| D16Ertd472e | 0.8080 | 0.0113 | 0.0364 | Akr1d1 | -0.7920 | 0.0000 | 0.0013 |
| Ell2 | 0.8066 | 0.0016 | 0.0117 | Acot11 | -0.7929 | 0.0016 | 0.0117 |
| Plin4 | 0.8059 | 0.0051 | 0.0227 | C1ra | -0.7951 | 0.0001 | 0.0039 |
| Stt3b | 0.8038 | 0.0002 | 0.0045 | Hnmt | -0.7971 | 0.0002 | 0.0045 |
| Hnrnpu | 0.8037 | 0.0003 | 0.0048 | Abcd3 | -0.7991 | 0.0000 | 0.0024 |
| Slc30a1 | 0.8026 | 0.0001 | 0.0029 | Slc35e2 | -0.7997 | 0.0005 | 0.0065 |
| Bcl3 | 0.8017 | 0.0101 | 0.0342 | Nox4 | -0.8014 | 0.0003 | 0.0052 |
| Mcrip1 | 0.8010 | 0.0002 | 0.0045 | Mpeg1 | -0.8025 | 0.0000 | 0.0024 |
| Slc45a3 | 0.8008 | 0.0014 | 0.0109 | Slc10a1 | -0.8031 | 0.0002 | 0.0045 |
| Zfand5 | 0.8008 | 0.0086 | 0.0310 | Klhdc7a | -0.8035 | 0.0009 | 0.0090 |
| Atl2 | 0.7991 | 0.0000 | 0.0025 | Fzd8 | -0.8050 | 0.0002 | 0.0046 |
| Dnaja3 | 0.7982 | 0.0018 | 0.0125 | Coq8a | -0.8055 | 0.0004 | 0.0058 |
| Avpi1 | 0.7978 | 0.0000 | 0.0010 | Acat3 | -0.8057 | 0.0006 | 0.0073 |
| Abca1 | 0.7946 | 0.0001 | 0.0035 | Spc25 | -0.8106 | 0.0014 | 0.0109 |
| Eif1 | 0.7919 | 0.0002 | 0.0043 | Daam1 | -0.8114 | 0.0000 | 0.0015 |
| Crls1 | 0.7916 | 0.0003 | 0.0049 | Mapk15 | -0.8149 | 0.0010 | 0.0091 |
| Igsf11 | 0.7907 | 0.0006 | 0.0071 | Hsd17b11 | -0.8161 | 0.0001 | 0.0028 |
| Slc7a2 | 0.7892 | 0.0031 | 0.0167 | Pde9a | -0.8168 | 0.0026 | 0.0153 |
| Hmgn2 | 0.7884 | 0.0006 | 0.0070 | Bst2 | -0.8176 | 0.0003 | 0.0052 |
| Nifk | 0.7864 | 0.0003 | 0.0049 | Hectd3 | -0.8216 | 0.0001 | 0.0035 |
| Iars | 0.7858 | 0.0001 | 0.0037 | Fcgr2b | -0.8231 | 0.0115 | 0.0369 |
| Derl3 | 0.7838 | 0.0097 | 0.0333 | Nadk2 | -0.8235 | 0.0031 | 0.0167 |
| Zfp617 | 0.7837 | 0.0017 | 0.0121 | Fam102a | -0.8241 | 0.0000 | 0.0023 |
| Eprs | 0.7835 | 0.0002 | 0.0044 | Sdc3 | -0.8244 | 0.0016 | 0.0117 |
| Pdia4 | 0.7818 | 0.0074 | 0.0282 | Abcc6 | -0.8247 | 0.0010 | 0.0090 |
| Palmd | 0.7788 | 0.0086 | 0.0308 | Shmt1 | -0.8259 | 0.0001 | 0.0038 |
| Adora1 | 0.7779 | 0.0008 | 0.0081 | Lipa | -0.8265 | 0.0000 | 0.0024 |
| Rhbdd1 | 0.7764 | 0.0003 | 0.0052 | Prodh2 | -0.8282 | 0.0002 | 0.0042 |
| Tob2 | 0.7763 | 0.0024 | 0.0146 | Hgd | -0.8291 | 0.0001 | 0.0036 |
| Xbp1 | 0.7761 | 0.0032 | 0.0170 | Gas2 | -0.8300 | 0.0005 | 0.0065 |
| Zkscan1 | 0.7757 | 0.0000 | 0.0013 | Ptprd | -0.8303 | 0.0011 | 0.0095 |
| Ugt1a9 | 0.7744 | 0.0020 | 0.0132 | Gm4951 | -0.8309 | 0.0000 | 0.0015 |
| Eppk1 | 0.7740 | 0.0028 | 0.0157 | Grb7 | -0.8364 | 0.0010 | 0.0091 |
| Angptl8 | 0.7732 | 0.0000 | 0.0024 | Qdpr | -0.8424 | 0.0006 | 0.0071 |
| Hsd17b13 | 0.7691 | 0.0107 | 0.0353 | Pcbd1 | -0.8441 | 0.0002 | 0.0045 |

| | | | | | | | |
|---|---|---|---|---|---|---|---|
| Tbpl1 | 0.7687 | 0.0017 | 0.0121 | Cabyr | -0.8481 | 0.0020 | 0.0132 |
| U2af1 | 0.7683 | 0.0001 | 0.0039 | Mthfd1 | -0.8521 | 0.0003 | 0.0049 |
| Tsc22d2 | 0.7675 | 0.0020 | 0.0132 | Phlda1 | -0.8541 | 0.0013 | 0.0103 |
| Prune1 | 0.7671 | 0.0036 | 0.0183 | Klf15 | -0.8566 | 0.0004 | 0.0056 |
| Afdn | 0.7630 | 0.0010 | 0.0090 | Gstp1 | -0.8578 | 0.0027 | 0.0156 |
| Tox | 0.7620 | 0.0121 | 0.0381 | Hagh | -0.8581 | 0.0014 | 0.0110 |
| Rras2 | 0.7576 | 0.0011 | 0.0095 | Zfyve21 | -0.8607 | 0.0002 | 0.0047 |
| Desi2 | 0.7567 | 0.0012 | 0.0100 | Acaa1b | -0.8611 | 0.0000 | 0.0015 |
| Lmo4 | 0.7556 | 0.0115 | 0.0368 | Ces2e | -0.8618 | 0.0003 | 0.0051 |
| Rpsa | 0.7550 | 0.0001 | 0.0028 | Osgin1 | -0.8643 | 0.0068 | 0.0269 |
| Nrg4 | 0.7522 | 0.0025 | 0.0148 | Ces1g | -0.8647 | 0.0008 | 0.0081 |
| App | 0.7510 | 0.0036 | 0.0183 | Slc2a2 | -0.8688 | 0.0000 | 0.0017 |
| Clock | 0.7499 | 0.0000 | 0.0014 | Gmnn | -0.8710 | 0.0009 | 0.0088 |
| Atf4 | 0.7486 | 0.0004 | 0.0060 | Akr1c12 | -0.8733 | 0.0002 | 0.0046 |
| Adcy9 | 0.7475 | 0.0005 | 0.0063 | Tcea3 | -0.8792 | 0.0006 | 0.0069 |
| Tubb6 | 0.7473 | 0.0014 | 0.0111 | Dnase1l3 | -0.8853 | 0.0053 | 0.0231 |
| Zfp869 | 0.7469 | 0.0020 | 0.0132 | Serpind1 | -0.8863 | 0.0001 | 0.0037 |
| 4921524J17Rik | 0.7465 | 0.0058 | 0.0243 | Abcc2 | -0.8864 | 0.0018 | 0.0125 |
| Crybg1 | 0.7421 | 0.0001 | 0.0032 | Dhfr | -0.8892 | 0.0001 | 0.0029 |
| C9orf72 | 0.7419 | 0.0074 | 0.0282 | Pnpla7 | -0.8904 | 0.0001 | 0.0028 |
| Eif3c | 0.7403 | 0.0002 | 0.0045 | Kcnk5 | -0.8925 | 0.0001 | 0.0033 |
| Retsat | 0.7400 | 0.0041 | 0.0199 | B4galt5 | -0.8948 | 0.0019 | 0.0128 |
| Slc38a2 | 0.7399 | 0.0010 | 0.0095 | Dbi | -0.8982 | 0.0001 | 0.0033 |
| Cln5 | 0.7385 | 0.0002 | 0.0040 | Prok2 | -0.8991 | 0.0002 | 0.0045 |
| Fam135a | 0.7374 | 0.0000 | 0.0017 | Dusp1 | -0.9000 | 0.0021 | 0.0137 |
| Kras | 0.7367 | 0.0000 | 0.0013 | Tmem25 | -0.9013 | 0.0008 | 0.0085 |
| Gramd1c | 0.7353 | 0.0002 | 0.0045 | Oaf | -0.9035 | 0.0026 | 0.0151 |
| Zmiz1 | 0.7343 | 0.0006 | 0.0071 | Sfxn5 | -0.9036 | 0.0002 | 0.0042 |
| Crip2 | 0.7341 | 0.0009 | 0.0087 | Sephs2 | -0.9042 | 0.0034 | 0.0177 |
| Mbnl2 | 0.7315 | 0.0001 | 0.0032 | Pdcd4 | -0.9206 | 0.0016 | 0.0117 |
| Rpl3 | 0.7278 | 0.0000 | 0.0017 | Zpr1 | -0.9215 | 0.0003 | 0.0052 |
| Fam107b | 0.7276 | 0.0019 | 0.0130 | Cyp2c67 | -0.9235 | 0.0005 | 0.0067 |
| Erlin1 | 0.7257 | 0.0003 | 0.0051 | Tob1 | -0.9296 | 0.0040 | 0.0196 |
| Kng2 | 0.7248 | 0.0002 | 0.0045 | Slc25a1 | -0.9339 | 0.0002 | 0.0043 |
| Trim24 | 0.7236 | 0.0014 | 0.0110 | Fbxo21 | -0.9358 | 0.0002 | 0.0042 |
| As3mt | 0.7235 | 0.0000 | 0.0014 | Lect2 | -0.9374 | 0.0096 | 0.0331 |
| Cyp26a1 | 0.7232 | 0.0019 | 0.0129 | Albfm1 | -0.9380 | 0.0014 | 0.0109 |
| Eif5 | 0.7224 | 0.0025 | 0.0148 | Irf7 | -0.9448 | 0.0039 | 0.0193 |
| Tns1 | 0.7222 | 0.0000 | 0.0024 | F830016B08Rik | -0.9484 | 0.0001 | 0.0028 |
| Ttpa | 0.7221 | 0.0024 | 0.0147 | Uox | -0.9555 | 0.0001 | 0.0039 |
| Pnrc1 | 0.7217 | 0.0003 | 0.0052 | Npr2 | -0.9590 | 0.0009 | 0.0089 |

| | | | | | | | |
|---|---|---|---|---|---|---|---|
| Epg5 | 0.7168 | 0.0001 | 0.0029 | Acat2 | -0.9605 | 0.0004 | 0.0056 |
| Nat8f2 | 0.7167 | 0.0041 | 0.0197 | Mug2 | -0.9639 | 0.0013 | 0.0103 |
| Gabarapl1 | 0.7164 | 0.0022 | 0.0137 | Tmsb4x | -0.9675 | 0.0013 | 0.0106 |
| Aig1 | 0.7140 | 0.0004 | 0.0055 | Arl6ip1 | -0.9723 | 0.0002 | 0.0042 |
| Eepd1 | 0.7128 | 0.0004 | 0.0057 | Mrpl18 | -0.9730 | 0.0023 | 0.0141 |
| Aars | 0.7124 | 0.0000 | 0.0025 | Klc4 | -0.9754 | 0.0007 | 0.0076 |
| Cp | 0.7116 | 0.0001 | 0.0029 | Abcg8 | -0.9773 | 0.0001 | 0.0036 |
| Tcea1 | 0.7072 | 0.0002 | 0.0044 | Tk1 | -0.9806 | 0.0000 | 0.0023 |
| Fosl2 | 0.7064 | 0.0023 | 0.0141 | Pecr | -0.9808 | 0.0000 | 0.0018 |
| Map4k4 | 0.7037 | 0.0003 | 0.0048 | Klkb1 | -0.9820 | 0.0002 | 0.0045 |
| Serinc3 | 0.7034 | 0.0007 | 0.0076 | Fdx1 | -0.9838 | 0.0000 | 0.0015 |
| Lrrc8d | 0.7033 | 0.0026 | 0.0152 | Spag7 | -0.9855 | 0.0004 | 0.0057 |
| Npas2 | 0.7008 | 0.0015 | 0.0112 | Col5a3 | -0.9856 | 0.0038 | 0.0189 |
| Cd302 | 0.6987 | 0.0000 | 0.0018 | Slc16a2 | -0.9862 | 0.0007 | 0.0079 |
| Icam1 | 0.6986 | 0.0171 | 0.0474 | Igfals | -0.9862 | 0.0000 | 0.0010 |
| Pxn | 0.6977 | 0.0004 | 0.0054 | Abhd14b | -0.9885 | 0.0003 | 0.0053 |
| Spg20 | 0.6975 | 0.0003 | 0.0050 | Pdk2 | -0.9953 | 0.0000 | 0.0015 |
| Amdhd1 | 0.6963 | 0.0034 | 0.0177 | Susd4 | -1.0009 | 0.0006 | 0.0069 |
| Gtf2ird1 | 0.6951 | 0.0002 | 0.0045 | Mafb | -1.0080 | 0.0004 | 0.0054 |
| Ip6k2 | 0.6951 | 0.0022 | 0.0137 | Ociad2 | -1.0090 | 0.0000 | 0.0025 |
| Slc20a1 | 0.6949 | 0.0004 | 0.0054 | Mmp15 | -1.0141 | 0.0004 | 0.0059 |
| Adgrl2 | 0.6946 | 0.0004 | 0.0057 | Acy3 | -1.0167 | 0.0000 | 0.0015 |
| Cdip1 | 0.6944 | 0.0021 | 0.0134 | Hykk | -1.0181 | 0.0005 | 0.0064 |
| Frat1 | 0.6924 | 0.0020 | 0.0131 | Ces1e | -1.0217 | 0.0001 | 0.0034 |
| H4c14 | 0.6923 | 0.0057 | 0.0241 | Akr1c19 | -1.0244 | 0.0012 | 0.0100 |
| Rock2 | 0.6923 | 0.0128 | 0.0396 | Bdh1 | -1.0249 | 0.0002 | 0.0045 |
| Rbm18 | 0.6917 | 0.0002 | 0.0039 | Stard4 | -1.0250 | 0.0001 | 0.0029 |
| Hnrnpf | 0.6906 | 0.0000 | 0.0025 | Hao1 | -1.0268 | 0.0002 | 0.0041 |
| Hnf4a | 0.6895 | 0.0029 | 0.0162 | Pcyt2 | -1.0391 | 0.0002 | 0.0045 |
| Fetub | 0.6867 | 0.0015 | 0.0113 | Myorg | -1.0398 | 0.0003 | 0.0052 |
| Cyp39a1 | 0.6840 | 0.0017 | 0.0121 | Ppara | -1.0487 | 0.0003 | 0.0052 |
| Slc10a2 | 0.6832 | 0.0001 | 0.0034 | Fads1 | -1.0615 | 0.0013 | 0.0105 |
| Rrbp1 | 0.6819 | 0.0030 | 0.0165 | Sucnr1 | -1.0619 | 0.0002 | 0.0040 |
| Ubb | 0.6808 | 0.0006 | 0.0072 | C4a | -1.0625 | 0.0039 | 0.0192 |
| Dgat2 | 0.6804 | 0.0097 | 0.0333 | Cebpa | -1.0638 | 0.0011 | 0.0096 |
| Serpinf2 | 0.6782 | 0.0032 | 0.0170 | Pltp | -1.0641 | 0.0005 | 0.0063 |
| Adtrp | 0.6772 | 0.0017 | 0.0121 | Rnase4 | -1.0707 | 0.0005 | 0.0066 |
| St3gal1 | 0.6749 | 0.0013 | 0.0105 | St13 | -1.0755 | 0.0001 | 0.0029 |
| Elk4 | 0.6734 | 0.0000 | 0.0025 | Spc24 | -1.0781 | 0.0000 | 0.0022 |
| B4galnt1 | 0.6727 | 0.0011 | 0.0098 | Dpys | -1.0810 | 0.0000 | 0.0022 |
| Rnf11 | 0.6721 | 0.0043 | 0.0203 | Nr1d2 | -1.0912 | 0.0001 | 0.0026 |

| | | | | | | | |
|---|---|---|---|---|---|---|---|
| Arhgef7 | 0.6709 | 0.0016 | 0.0118 | Cyp2d40 | -1.0992 | 0.0000 | 0.0015 |
| Zfp110 | 0.6692 | 0.0017 | 0.0121 | Lipg | -1.1041 | 0.0012 | 0.0102 |
| Cebpb | 0.6685 | 0.0041 | 0.0197 | Nmrk1 | -1.1045 | 0.0001 | 0.0036 |
| Acnat1 | 0.6685 | 0.0002 | 0.0042 | Ugt2b38 | -1.1066 | 0.0001 | 0.0039 |
| Ikbkg | 0.6663 | 0.0008 | 0.0083 | Cyp7b1 | -1.1115 | 0.0007 | 0.0080 |
| Gadd45g | 0.6655 | 0.0173 | 0.0477 | Slc25a23 | -1.1137 | 0.0044 | 0.0206 |
| Cebpg | 0.6646 | 0.0029 | 0.0163 | Slc17a2 | -1.1151 | 0.0003 | 0.0048 |
| Gpcpd1 | 0.6641 | 0.0142 | 0.0423 | Ifi27 | -1.1174 | 0.0022 | 0.0138 |
| Nudt18 | 0.6634 | 0.0021 | 0.0134 | Selenbp1 | -1.1208 | 0.0006 | 0.0068 |
| Skil | 0.6621 | 0.0017 | 0.0121 | Serpina6 | -1.1308 | 0.0003 | 0.0050 |
| Cast | 0.6617 | 0.0005 | 0.0066 | Cyb5r3 | -1.1358 | 0.0000 | 0.0017 |
| Sh3pxd2a | 0.6606 | 0.0006 | 0.0068 | Car5a | -1.1419 | 0.0001 | 0.0037 |
| Myh10 | 0.6585 | 0.0001 | 0.0031 | Ces1f | -1.1491 | 0.0001 | 0.0031 |
| Nek6 | 0.6581 | 0.0003 | 0.0052 | Gpi1 | -1.1507 | 0.0001 | 0.0037 |
| Asl | 0.6554 | 0.0116 | 0.0371 | Onecut1 | -1.1546 | 0.0002 | 0.0048 |
| Ifitm2 | 0.6551 | 0.0003 | 0.0052 | Ghr | -1.1637 | 0.0005 | 0.0065 |
| Mthfr | 0.6538 | 0.0016 | 0.0117 | Hsd3b3 | -1.1645 | 0.0001 | 0.0035 |
| Rsc1a1 | 0.6538 | 0.0041 | 0.0198 | Akr1c20 | -1.1735 | 0.0002 | 0.0045 |
| Anapc16 | 0.6525 | 0.0003 | 0.0052 | Slc25a13 | -1.1783 | 0.0001 | 0.0031 |
| Bco1 | 0.6507 | 0.0051 | 0.0226 | Tef | -1.1826 | 0.0002 | 0.0042 |
| Rbp4 | 0.6502 | 0.0010 | 0.0091 | Pbld2 | -1.1956 | 0.0000 | 0.0025 |
| Syt12 | 0.6490 | 0.0046 | 0.0214 | Nrep | -1.1974 | 0.0001 | 0.0027 |
| Frmd4b | 0.6469 | 0.0028 | 0.0158 | Acbd5 | -1.1980 | 0.0000 | 0.0010 |
| Lsm14a | 0.6465 | 0.0002 | 0.0041 | Sec14l2 | -1.1994 | 0.0000 | 0.0015 |
| Nt5c2 | 0.6433 | 0.0005 | 0.0061 | Nxpe2 | -1.1995 | 0.0006 | 0.0072 |
| Cps1 | 0.6430 | 0.0078 | 0.0290 | Alad | -1.2124 | 0.0000 | 0.0021 |
| Pcdh1 | 0.6430 | 0.0007 | 0.0074 | Pygl | -1.2186 | 0.0000 | 0.0016 |
| Slc34a2 | 0.6416 | 0.0038 | 0.0190 | Ces3b | -1.2365 | 0.0005 | 0.0063 |
| Cldn14 | 0.6410 | 0.0003 | 0.0049 | Plcxd2 | -1.2441 | 0.0001 | 0.0026 |
| Scara5 | 0.6408 | 0.0138 | 0.0417 | C9 | -1.2579 | 0.0086 | 0.0310 |
| Rai14 | 0.6399 | 0.0001 | 0.0036 | Ak2 | -1.2718 | 0.0001 | 0.0036 |
| Gm12394 | 0.6396 | 0.0017 | 0.0121 | Car3 | -1.2833 | 0.0001 | 0.0028 |
| Gata6 | 0.6383 | 0.0011 | 0.0095 | Alas2 | -1.2863 | 0.0001 | 0.0029 |
| Sstr2 | 0.6382 | 0.0027 | 0.0155 | Pdxk | -1.2911 | 0.0000 | 0.0016 |
| Mindy1 | 0.6375 | 0.0019 | 0.0128 | Hpgd | -1.2992 | 0.0000 | 0.0010 |
| Shtn1 | 0.6369 | 0.0000 | 0.0021 | Slc25a25 | -1.3136 | 0.0008 | 0.0081 |
| Pole4 | 0.6357 | 0.0000 | 0.0025 | Srd5a1 | -1.3144 | 0.0001 | 0.0026 |
| Srsf1 | 0.6347 | 0.0003 | 0.0048 | Narf | -1.3253 | 0.0000 | 0.0021 |
| Nfia | 0.6347 | 0.0002 | 0.0042 | Apol9b | -1.3307 | 0.0001 | 0.0031 |
| Stk40 | 0.6331 | 0.0024 | 0.0147 | Adh4 | -1.3401 | 0.0003 | 0.0051 |
| Gadd45b | 0.6323 | 0.0019 | 0.0130 | Cyp2u1 | -1.3534 | 0.0025 | 0.0150 |

| | | | | | | | |
|---|---|---|---|---|---|---|---|
| Kctd15 | 0.6314 | 0.0029 | 0.0163 | Hspa8 | -1.3567 | 0.0001 | 0.0026 |
| Ap5s1 | 0.6308 | 0.0052 | 0.0229 | Abcg5 | -1.3600 | 0.0000 | 0.0010 |
| Hspa9 | 0.6304 | 0.0000 | 0.0023 | Gulo | -1.3631 | 0.0010 | 0.0094 |
| Rrp36 | 0.6297 | 0.0003 | 0.0049 | Col27a1 | -1.3842 | 0.0001 | 0.0036 |
| Ddx52 | 0.6282 | 0.0025 | 0.0149 | Hmgcr | -1.3924 | 0.0000 | 0.0017 |
| Smoc1 | 0.6264 | 0.0022 | 0.0139 | Sord | -1.3982 | 0.0001 | 0.0036 |
| Myd88 | 0.6260 | 0.0016 | 0.0118 | Usp2 | -1.4151 | 0.0001 | 0.0033 |
| Mettl1 | 0.6249 | 0.0011 | 0.0096 | Acss2 | -1.4323 | 0.0004 | 0.0058 |
| Chd1 | 0.6240 | 0.0027 | 0.0156 | Apol7a | -1.4428 | 0.0000 | 0.0015 |
| Arhgdia | 0.6230 | 0.0000 | 0.0023 | Elovl6 | -1.4520 | 0.0002 | 0.0043 |
| Psmd8 | 0.6228 | 0.0002 | 0.0045 | Hes6 | -1.4648 | 0.0002 | 0.0042 |
| Bcl10 | 0.6224 | 0.0012 | 0.0103 | Cxcl12 | -1.4828 | 0.0000 | 0.0014 |
| Sars | 0.6222 | 0.0000 | 0.0019 | Nr1d1 | -1.4873 | 0.0002 | 0.0040 |
| Rpl12 | 0.6201 | 0.0001 | 0.0036 | Nat8f1 | -1.5084 | 0.0000 | 0.0018 |
| Psat1 | 0.6178 | 0.0008 | 0.0081 | Sult2a8 | -1.5193 | 0.0000 | 0.0013 |
| Zbtb21 | 0.6170 | 0.0047 | 0.0217 | Thrsp | -1.5364 | 0.0002 | 0.0043 |
| Tlcd4 | 0.6148 | 0.0166 | 0.0465 | Cat | -1.5466 | 0.0001 | 0.0037 |
| Oga | 0.6143 | 0.0025 | 0.0148 | Fabp2 | -1.6196 | 0.0001 | 0.0037 |
| Ablim3 | 0.6138 | 0.0141 | 0.0421 | Abca8a | -1.6280 | 0.0002 | 0.0039 |
| Rela | 0.6126 | 0.0059 | 0.0246 | Gys2 | -1.6306 | 0.0001 | 0.0031 |
| Stx6 | 0.6125 | 0.0001 | 0.0039 | Ldlr | -1.6413 | 0.0000 | 0.0015 |
| Stat3 | 0.6118 | 0.0015 | 0.0112 | Aacs | -1.6465 | 0.0000 | 0.0025 |
| Errfi1 | 0.6096 | 0.0009 | 0.0088 | Hsd3b1 | -1.6470 | 0.0013 | 0.0103 |
| Huwe1 | 0.6062 | 0.0004 | 0.0057 | Pklr | -1.6496 | 0.0000 | 0.0015 |
| Zscan21 | 0.6056 | 0.0066 | 0.0264 | Fads2 | -1.6514 | 0.0002 | 0.0047 |
| Wrnip1 | 0.6050 | 0.0005 | 0.0066 | C6 | -1.6849 | 0.0000 | 0.0022 |
| Trim2 | 0.6048 | 0.0046 | 0.0214 | Ugt2b1 | -1.6874 | 0.0018 | 0.0123 |
| Cda | 0.6028 | 0.0031 | 0.0167 | Elovl2 | -1.7003 | 0.0000 | 0.0014 |
| Septin11 | 0.6024 | 0.0095 | 0.0328 | Rgn | -1.7067 | 0.0001 | 0.0031 |
| Tm7sf3 | 0.6020 | 0.0003 | 0.0052 | Akr1c14 | -1.7409 | 0.0000 | 0.0013 |
| Gsta5 | 0.6016 | 0.0081 | 0.0296 | Slco1b2 | -1.7440 | 0.0003 | 0.0048 |
| Dynll1 | 0.6000 | 0.0001 | 0.0031 | Slc22a28 | -1.8364 | 0.0002 | 0.0045 |
| Myl12a | 0.5988 | 0.0001 | 0.0029 | Inmt | -1.9206 | 0.0000 | 0.0015 |
| Mocs2 | 0.5987 | 0.0002 | 0.0045 | G6pc | -1.9356 | 0.0000 | 0.0015 |
| Slc25a39 | 0.5957 | 0.0047 | 0.0217 | Akr1c6 | -1.9502 | 0.0001 | 0.0026 |
| Kpnb1 | 0.5933 | 0.0025 | 0.0148 | Fabp1 | -1.9668 | 0.0000 | 0.0019 |
| Mrps18b | 0.5857 | 0.0015 | 0.0116 | Ttc39c | -1.9957 | 0.0000 | 0.0015 |
| Ints12 | 0.5850 | 0.0011 | 0.0095 | Etnppl | -2.0175 | 0.0018 | 0.0126 |
| Aco1 | -0.5847 | 0.0001 | 0.0039 | Mup13 | -2.0266 | 0.0000 | 0.0024 |
| Pilrb1 | -0.5851 | 0.0075 | 0.0284 | Scd1 | -2.0346 | 0.0004 | 0.0056 |
| Lrpprc | -0.5858 | 0.0026 | 0.0154 | Acly | -2.3370 | 0.0001 | 0.0030 |

| | | | | | | | |
|---|---|---|---|---|---|---|---|
| Ttc38 | -0.5865 | 0.0001 | 0.0029 | Fasn | -2.3624 | 0.0000 | 0.0017 |
| Pdcd6 | -0.5868 | 0.0007 | 0.0078 | Hsd3b2 | -2.4061 | 0.0005 | 0.0068 |
| 1700012A03Rik | -0.5870 | 0.0081 | 0.0296 | Insig1 | -2.4495 | 0.0000 | 0.0010 |
| Rab43 | -0.5874 | 0.0004 | 0.0060 | Slco1a1 | -2.4603 | 0.0000 | 0.0023 |
| Gatm | -0.5874 | 0.0022 | 0.0138 | Alas1 | -2.4630 | 0.0001 | 0.0028 |
| Nr3c2 | -0.5876 | 0.0029 | 0.0162 | Aox3 | -2.5138 | 0.0002 | 0.0048 |
| H2-Q1 | -0.5880 | 0.0004 | 0.0060 | Nudt7 | -2.5812 | 0.0001 | 0.0039 |
| Rassf7 | -0.5891 | 0.0002 | 0.0047 | Cyp4a12a | -2.8309 | 0.0019 | 0.0128 |
| Pfkl | -0.5902 | 0.0027 | 0.0155 | Dbp | -2.8899 | 0.0000 | 0.0017 |
| Erbb3 | -0.5904 | 0.0002 | 0.0040 | Srebf1 | -2.9280 | 0.0002 | 0.0045 |
| Plcg1 | -0.5906 | 0.0005 | 0.0063 | Keg1 | -3.0059 | 0.0001 | 0.0028 |
| Mcoln1 | -0.5910 | 0.0000 | 0.0020 | Hsd3b5 | -3.1734 | 0.0000 | 0.0011 |
| Anxa7 | -0.5910 | 0.0000 | 0.0015 | Upp2 | -3.2705 | 0.0000 | 0.0024 |
| Phactr2 | -0.5911 | 0.0168 | 0.0470 | Cyp7a1 | -3.5145 | 0.0000 | 0.0018 |
